# Preparation, physicochemical characterization, and bioactivity evaluation of berberine-entrapped albumin nanoparticles

**DOI:** 10.1038/s41598-022-21568-8

**Published:** 2022-10-19

**Authors:** Fatema A. Younis, Samar R. Saleh, Sahar S. Abd El-Rahman, Al-Sayeda A. Newairy, Maha A. El-Demellawy, Doaa A. Ghareeb

**Affiliations:** 1 Bio-Screening and Preclinical Trial Lab, Biochemistry Department, Faculty of Science, Alexandria University, Alexandria, Egypt; 2 Department of Pathology, Faculty of Veterinary Medicine, Cairo University, Giza, Egypt; 3 Biochemistry Department, Faculty of Science, Alexandria University, Alexandria, Egypt; 4 Pharmaceutical and Fermentation Industries Development Centre (PFIDC), The City of Scientific Research and Technological Applications (SRTA-City), Borg Al-Arab, Alexandria, Egypt; 5 Medical Biotechnology Department, GEBRI, SRTA-City, New Borg El-Arab City, Alexandria, Egypt

**Keywords:** Biochemistry, Biological techniques, Biotechnology, Drug discovery

## Abstract

Berberine (BBR) is an isoquinoline alkaloid with several clinical therapeutic applications. Its low water solubility, absorption, and cellular bioavailability diminish BBR's therapeutic efficacy. In this study, BBR was encapsulated into bovine serum albumin nanoparticles (BSA NPs) core to reduce BBR limitations and enhance its clinical therapeutic properties. Several physicochemical characterization tools, such as Dynamic Light Scattering and Ultraviolet–Visible spectroscopic measurements, field emission transmission electron microscopy surface morphology, Fourier transforms infrared spectroscopy, thermal stability analysis, and releasing studies, were used to evaluate the BBR-BSA NPs. Compared to BBR, BBR-BSA nanoparticles demonstrated superior free radical scavenging and antioxidant capacities, anti-hemolytic and anticoagulant efficacies, and antimicrobial activities, as demonstrated by the findings of the in vitro studies. Furthermore, a stressed pancreatic rat model was induced using a high-fat, high-sucrose diet plus carbon tetrachloride injection. The in vivo results revealed that BBR-BSA NPs substantially restored peripheral glucose metabolism and insulin sensitivity. Oral administration of BBR-BSA NPs also improved pancreatic β-cells homeostasis, upregulated pancreatic antioxidant mechanisms, inhibited oxidants generation, and attenuated oxidative injury in the stressed pancreatic tissues. In conclusion, our in vitro and in vivo results confirmed that BBR-BSA NPs demonstrated more potent antioxidant properties and restored pancreatic homeostasis compared to BBR.

## Introduction

Oxidative stress-related diseases, such as cancer, diabetes, neurodegeneration, and inflammation usually begin with the overproduction of reactive oxygen/nitrogen species in the living cells. The oxidative stress response is the imbalance between the oxidants (free radicals) production and the antioxidant defense system, impairs DNA repairing systems, induces cellular lipid peroxidation, and leads to the development of oxidative stress-related diseases^1,2^. The cellular enzymatic antioxidants include superoxide dismutase (SOD), glutathione peroxidase (GSHPx), and glutathione-S-transferase (GST), while the cellular non-enzymatic antioxidants represent reduced glutathione (GSH) and vitamin C, E and D^3^. However, superoxide anion (O_2_^−^), hydrogen peroxide (H_2_O_2_), and hydroxyl ions (HO^−^) are considered highly reactive free radicals. The cellular antioxidant modulators, including SOD and GSHPx enzymes, promote the protective reactions in the cells and modulate oxidative stress responses^3,4^.

Green chemistry is a green biosynthesis that reduces cytotoxicity and increases antioxidant efficacy, tissue selectivity, and therapeutic applications of natural products. These products include several bioactive compounds with potent clinical pharmacological properties^5^. Berberine (BBR, C_20_H_19_NO_5_) is a derivative of isoquinoline alkaloid compound found in the root, rhizome, stem, fruit, and bark of various species of medicinal plants^6,7^. BBR formulations are extensively used to treat inflammation, insulin resistance, diabetes^8^, neurodegeneration, and liver diseases^9,10^. It also has several pharmacological actions, including antimicrobial, hypoglycemic, hypolipidemic, anti-obesity^7,11^, wound healing^12^, pro-apoptotic, anticancer, hepato- and neuro-protective, and anti-osteoporosis^13,14^. Some of these activities have been attributed to the potent antioxidant activity of BBR.

Berberine chloride, the salt form of BBR, is a quaternary ammonium isoquinoline alkaloid compound (C_20_H_18_ClNO_4_) that forms yellow needle-like crystals^8^. The poor water solubility of BBR increases the rates of its self-aggregation, local dissolution, and metabolism, as well as its elimination and clearance rates from the body. Therefore, its intestinal absorption activity, cellular bioavailability, and clinical therapeutic applications are reduced^15,16^. The oral bioavailability of BBR may be < 1%^17^ or < 5%^18^. A high dose of BBR (100–250 mg kg^−1^ day^−1^ for animals, 900–1500 mg day^−1^ for humans) is needed to target the beneficial therapeutic activity^17^. Anorexia, stomach upset, diarrhea, and/or constipation have been reported as side effects of high doses of BBR, which can eliminate the therapeutic properties of BBR^17^.

Many techniques were used to solve the solubility problems of BBR, such as particle-size reduction, solid dispersion, and polymeric nano-particulate drug delivery form. Nanoparticles (NPs) are smaller particles than traditional drug particles by increasing the drug surface area^19^. Albumin, a drug delivery tool, has a selective delivery potential, bioavailable, biocompatible, biodegradable, and non-toxic properties^20^. Additionally, albumin is metabolized in vivo to safe byproducts^21^. These byproducts are water-soluble and not immunogenic^22^. Albumin, an attractive polymer, has different drug binding sites. Human and bovine serum albumin (HSA and BSA) are the main types widely used in biopharmaceutical applications^23,24^. BSA has a molecular weight of 69,323 Da, and its isoelectric point is 4.7–4.9 in water at 25 °C^21,25^. BSA can be thermally stable over a pH range of 4 to 9. It can transport nutrients to different cells, solubilize the hydrophobic molecules, and enhance the aqueous solubility of the hydrophobic drugs in plasma^14^. In addition, it can be encapsulated into the core of BSA NPs for efficient therapeutic delivery^14^. Furthermore, BSA, a nano-carrier, is used to deliver several bioactive compounds due to its medical status, richness, low cost, and ease of purification^24^. Moreover, BSA NPs have many functional carboxylic and amino groups on their surface. These groups increase the covalent bonds between the BSA NPs and the molecules of the natural products^25^. BSA NPs can also eliminate cytotoxicity in clinical applications by reducing the drug's therapeutic dosages^22^. BSA NPs, a nano-particulate drug delivery tool, has been improved the solubility problems, bioavailability, selectivity, and therapeutic applications of several bioactive compounds^14,22,24^. In the present study, BBR-BSA NPs were synthesized to improve the therapeutic efficacy of BBR. The physicochemical characterizations of BBR-BSA NPs were determined using Dynamic Light Scattering (DLS) analysis, Ultraviolet–Visible (UV–Vis) spectroscopic measurements, Fourier transforms infrared (FTIR) spectroscopy, Field emission- transmission electron microscopy (FE-TEM), and thermal stability analysis. In addition, the in vitro and in vivo antioxidant properties of BBR-BSA NPs and its hypoglycemic and pancreatic protective effects were evaluated compared to BBR.

## Results

### Physicochemical characterizations of BBR-BSA NPs and BSA NPs

#### Evaluation of the particle size, polydispersity indices (PDI), zeta potential, surface morphology, encapsulation efficiency (EE%), loading capacity (LC%), and synthesis yield (SY%) of BBR-BSA NPs and BSA NPs

**Figure 1:**
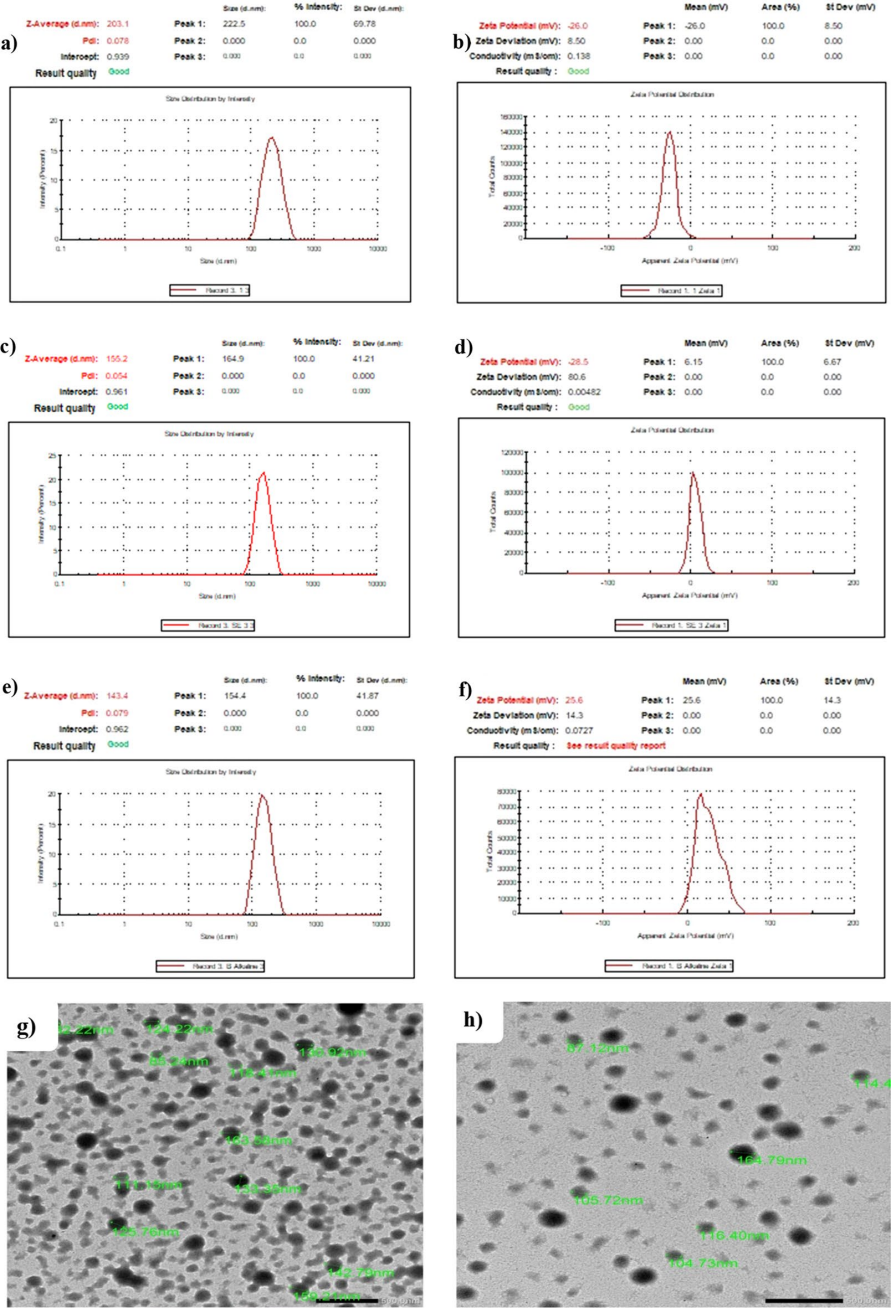
The hydrodynamic particle size diameter (nm), PDI, and zeta potential (mV) of neutral BBR-BSA NPs (**a**, **b**); alkaline BBR-BSA NPs (**c**, **d**); alkaline BSA NPs (**e**, **f**). The TEM photomicrographs of alkaline BBR-BSA NPs, magnification = 10,000 × (**g**); alkaline BSA NPs, magnification = 12,000 × (**h**). (Scale bar is 500 nm).

The BBR-BSA NPs were prepared using the desolvation method under neutral and alkaline conditions, as depicted in Supplementary Table S1. The average hydrodynamic particle size, PDI, and zeta potential were 202 ± 1.2 nm, 0.088 ± 0.019 and -26.65 ± 0.92 mV for neutral BBR-BSA NPs; 155 ± 0.6 nm, 0.060 ± 0.015 and -29.15 ± 0.92 mV for alkaline BBR-BSA NPs and 142 ± 1.5 nm, 0.086 ± 0.007 and 26.20 ± 0.85 mV for alkaline BSA NPs (Fig. 1a–f). Based on these results, we selected alkaline BBR-BSA NPs for further characterization. The surface morphology of alkaline BBR-BSA NPs and BSA NPs with smooth surfaces was estimated using FE-TEM micrographs demonstrating spherical, regular, and uniform shapes (Fig. 1g,h). Furthermore, FE-TEM micrographs demonstrated the alkaline BBR-BSA NPs and BSA NPs size diameters were 125 ± 23.60 nm and 159 ± 31.19 nm, respectively. Moreover, alkaline BBR-BSA NPs has efficient properties, as indicated by 18.8 ± 0.1 mg BBR-loading content, 208.1 ± 0.2 mg NPs content, 92.9 ± 0.6% EE, 9.1 ± 0.1% LC, and 94.4 ± 0.1% SY. In addition, 195.3 ± 0.1 mg NPs content and 97.3 ± 0.1% SY of the prepared alkaline BSA NPs.

#### The FTIR characteristic spectra of the synthesized BBR-BSA NPs and BSA NPs

**Figure 2:**
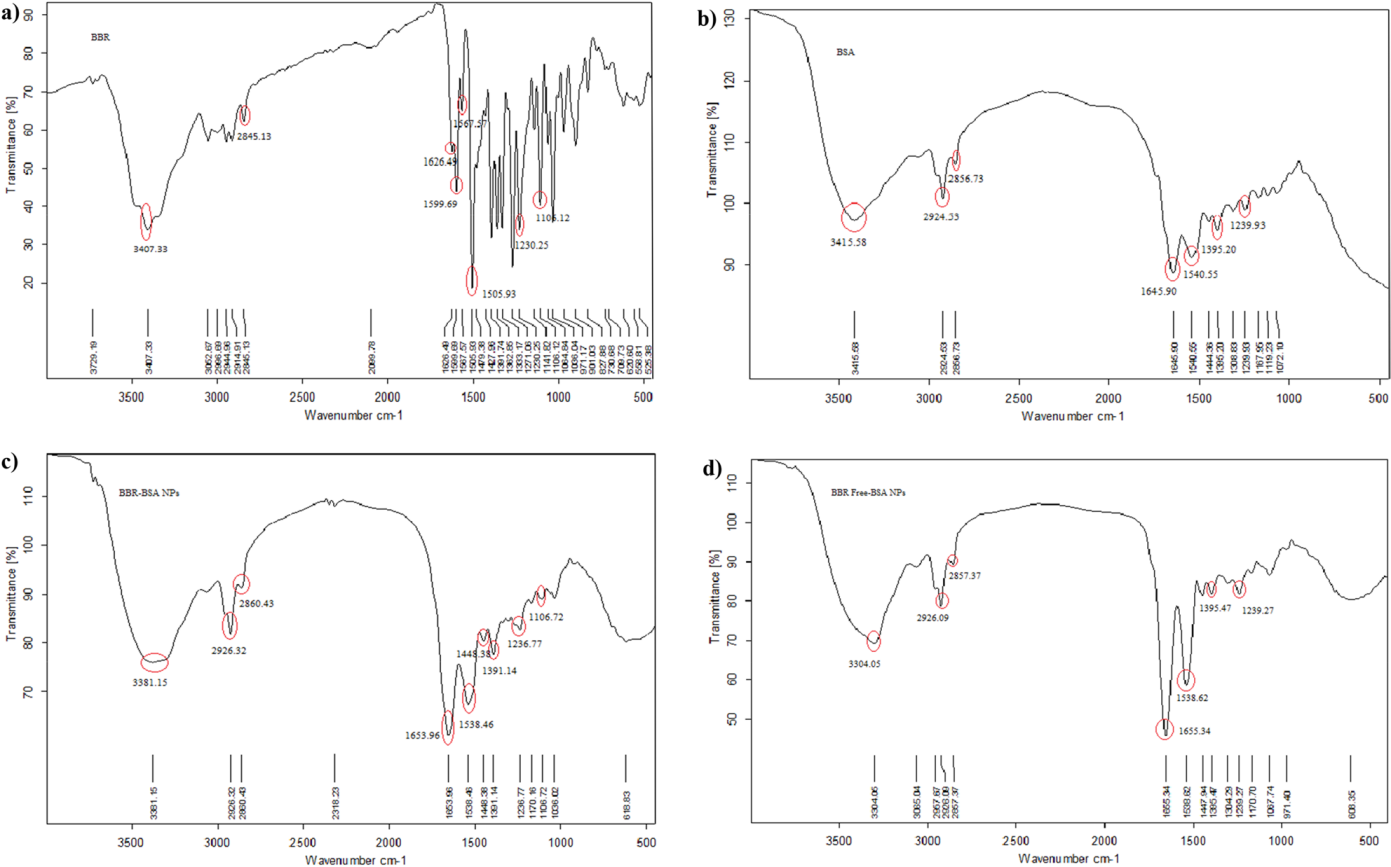
The FTIR characteristic spectra of standard BBR-chloride (**a**), crystalline BSA (**b**), BBR-BSA NPs (**c**), and BSA NPs (**d**).

The characteristic FTIR spectra of standard BBR-chloride, crystalline BSA, BBR-BSA NPs, and BSA NPs are demonstrated in Fig. 2a–d. Significant crystalline BSA peaks were observed at 3415.58 cm^−1^ (O–H stretching vibration band), 2924.53 cm^−1^ (amide A band, related to N–H stretching vibration), 2856.73 cm^−1^ (amide B band, related to N–H stretching vibration of NH_3_^+^ free ions), 1645.90 cm^−1^ (amide I band related to C=O stretching vibration of the peptide linkages, indicating the secondary structure of the BSA globular protein), 1540.55 cm^−1^ (amide II band considered as the standard protein peak, which referred to the coupling of the in-plane bending vibrations of N–H and the stretching vibrations of C–N and C–C), 1444.36–1395.20 cm^−1^ (CH_2_ bending groups, indicated the C–H bending vibrations), and 1239.93 cm^−1^ (amide III bands, related to the combination of the in-plane N–H bending vibrations and the C–N stretching vibrations) (Fig. 2b). There was a relative change in the band intensities of the different functional characteristic peaks of crystalline BSA (Fig. 2b), compared to the FTIR spectra of BBR-BSA NPs and BSA NPs (Fig. 2c,d and Supplementary Table S2). It was induced by the chemical interactions and the conformational changes between the BSA molecules following the formulation and preparation of the NPs (Fig. 2b–d). Significant peaks of standard BBR-chloride were observed at 3407.33 cm^−1^ (O–H stretching vibration), 3052.67 cm^−1^ (hydrophilic quaternary ammonium (N^+^) group, which was related to the binding of this group to four hydrophobic aromatic heterocyclic hydrocarbons groups through covalent C-N bonds), 2944.96 cm^−1^ (saturated C–H stretching), 2845.13 cm^−1^ (–OCH_3_ methoxyl group), 1626.49 cm^−1^ (heterocyclic amines as a C–N band), 1599.69 cm^−1^ (heterocyclic amines as a C=N^+^ quaternary iminium ion stretching vibration band), 1567.57 cm^−1^ (C=C bending vibration in the aromatic ring), 1505.93 cm^−1^ (furyl group and C=C stretching vibrations band), 1479.38–1427.96 cm^−1^ (–CH_2_– methylene), 1391.74–1362.85 cm^−1^ (–CH_3_ stretching vibrations), 1333.17–1036.04 cm^−1^ (C–O–C bonding), 971.17–730.68 cm^−1^ (=C–H “OOP” out of plane), and 620.60—558.81 cm^−1^ (chloride as halide band) (Fig. 2a). The FTIR spectrum of BBR-BSANPs demonstrated significant peaks at 2926.32 cm^−1^, 1653.96 cm^−1^, and 1538.46 cm^−1^, indicating the presence of the amide A, amide I, and amide II bands of BSA (Fig. 2c and Supplementary Table S2). The characteristics peaks at 3381.15 cm^−1^, 2860.43 cm^−1^, and 618.83 cm^−1^ confirmed the presence of the OH stretching vibrations, –OCH3 methoxyl groups, and the chloride ions of the entrapped BBR in the BSA core, respectively (Fig. 2c and Supplementary Table S3).

#### The thermogravimetric analysis of the BBR-BSA NPs and BSA NPs

**Figure 3:**
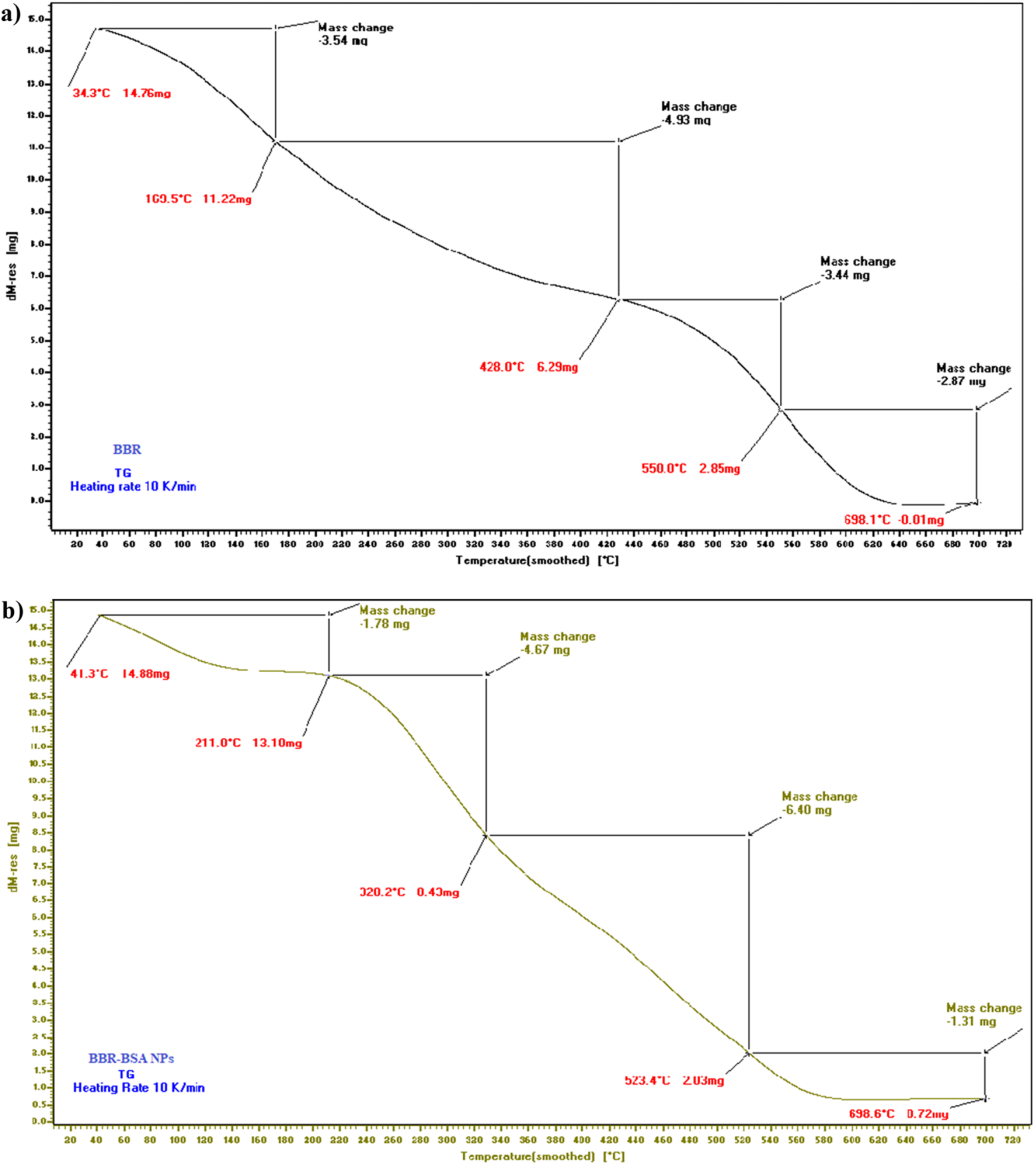
The TGA thermograms of standard BBR-chloride (**a**) and BBR-BSA NPs (**b**).

The thermal gravimetric analysis (TGA) thermograms revealed the variations in the mass degradation rates of standard BBR-chloride, crystalline BSA, and BBR-BSA NPs related to the increased temperature (Fig. 3 and Supplementary Fig. S1a). Compared to the thermogram of standard BBR-chloride at 170 °C, the mass degradation rate of the TGA thermogram of BBR-BSA NPs (Fig. 3b) revealed a significant decrease in the mass degradation rate before 210 °C (Fig. 3a). After formulation, the thermal stability of the entrapped BBR was increased.

**Figure 4:**
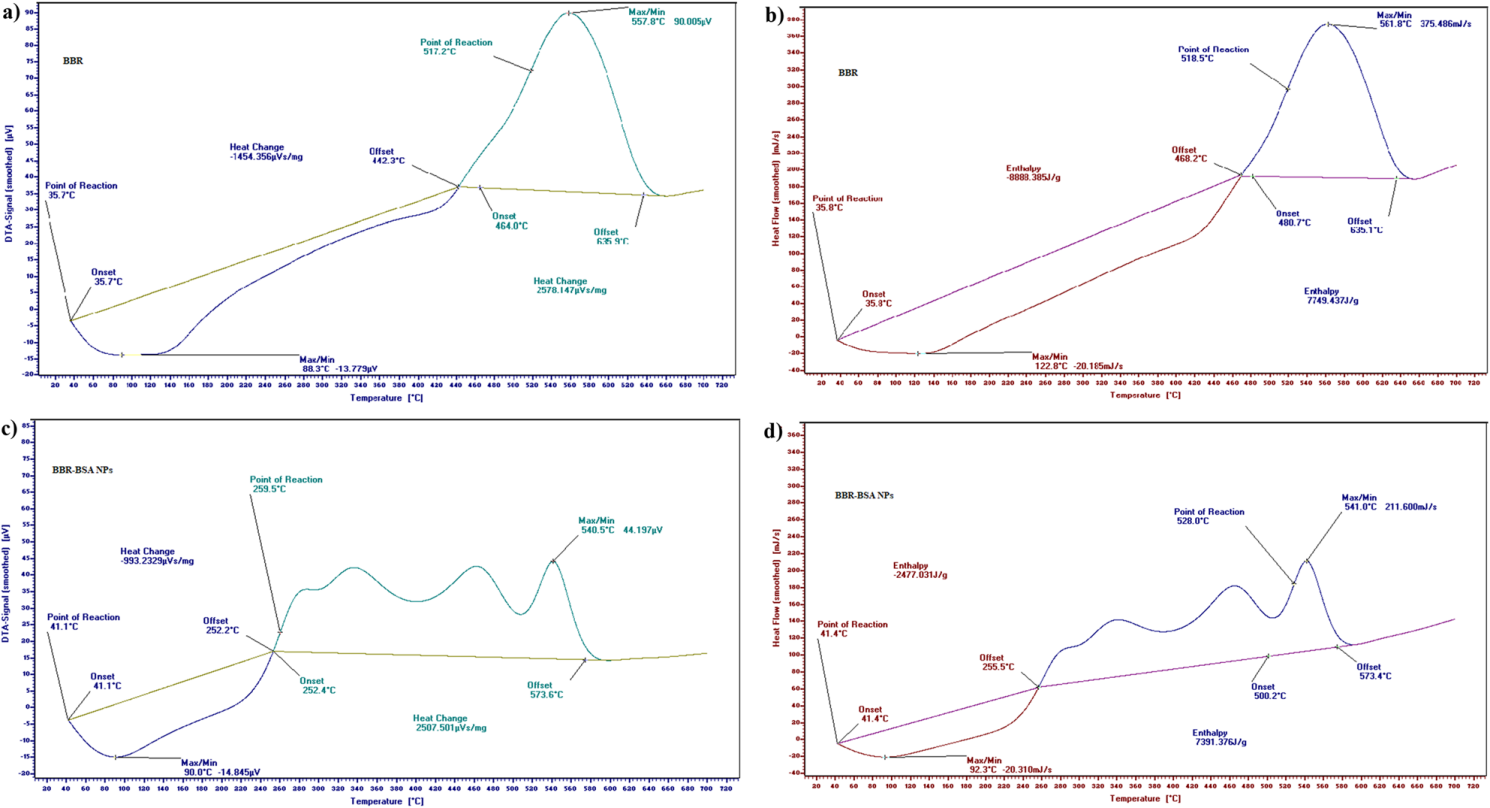
Thermograms of standard BBR-chloride and BBR-BSA NPs DTA (**a**, **c**), and DSC (**b**, **d**), respectively.

The Differential thermal analysis (DTA) and differential scanning calorimetry (DSC) thermograms of standard BBR-chloride demonstrated two points for reaction, the first at 35.7 °C (DTA); 35.8 °C (DSC), and the second at 517.2 °C (DTA); 518.5 °C (DSC) (Fig. 4a,b). In the temperature range of 535.7–634.2 °C (DTA); 520.7–638.5 °C (DSC), a large, sharp, and slender melting endothermic peak was observed. It indicated the crystalline nature of BBR-chloride (Fig. 4a,b). The DTA/DSC thermograms of BSA revealed two points for reaction, the first at 41.5 °C (TGA); 41.5°C (DSC), and the second at 549.2 °C (DTA); 549.8 °C (DSC) (Supplementary Fig. S1b,c). A large sharp, slightly narrow melting endothermic peak was demonstrated at the 535.7–634.2 °C (DTA); 520.7–638.5 °C (DSC) temperature range (Supplementary Fig. S1b,c). The DTA/DSC thermograms of BBR-BSA NPs revealed two different points for reaction, the first at 41.1 °C (DTA); 41.4 °C (DSC), and the second at 259.5 °C (DTA); 528.0 °C (DSC) (Fig. 4c,d). These points varied from the points of reaction of standard BBR-chloride and crystalline BSA. The DTA/DSC thermograms of BBR-BSA NPs have also demonstrated a decrease in heat change values from -1454.36 and 2578.15 µVs/mg to -993.23 and 2507.50 µV/mg (Fig. 4c) and a reduction in the values of the enthalpy from -8888.39 and 7749.44 J/g to − 2477.03 and 7391.38 J/g (Fig. 4d), compared to the DTA/DSC thermograms of standard BBR-chloride (Fig. 4a,b).

### In vitro studies

#### Evaluation of the BBR releasing rates (%)

**Figure 5:**
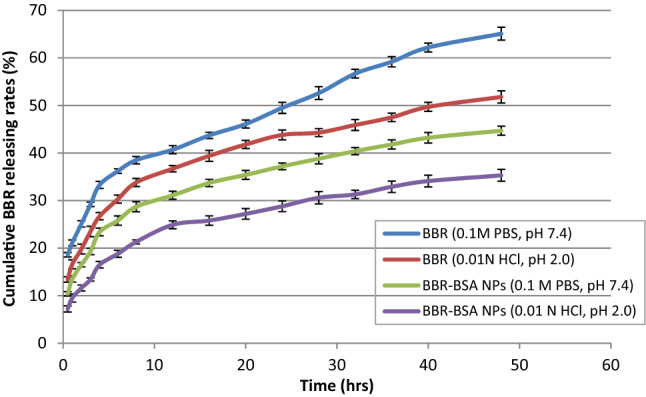
The BBR-releasing rate after 48 h. Data values are expressed as means ± SD (n = 5).

Figure 5 shows a significant decrease in the releasing and local dissolution rates of BBR-BSA NPs compared to BBR in different dialysis media. Simulated intestinal fluid (SIF, pH 7.4) and simulated gastric fluid (SGF, pH 2) was used. The SGF medium reduced the releasing and local dissolution rates of BBR and BBR-BSA NPs compared to their rates in the SIF medium. After 48 h, the releasing rates of the entrapped BBR (BBR-BSA NPs) were only about 40 and 35% in SIF and SGF, compared to about 65 and 50% release of BBR, respectively.

#### Estimation of the free radicals scavenging activities and the total antioxidant capacities of BBR-BSA NPs and BSA NPs

The antioxidant properties of the tested compounds increased in a concentration-dependent manner (Supplementary Figs. S2–S9). The radical scavenging activities showed that BBR-BSA NPs significantly (*P* < 0.05) inhibited NO^·^ (68.77%), H_2_O_2_ (94.71%), O_2_^−^ (64.22%), OH^−^ (98.81%), DPPH (98.91%), liver TBARS (97.44%), and brain TBARS (90.11%) at a concentration of 1.8 mg/mL (Supplementary Table S4), with IC_50_ values of 0.71, 0.64, 1.21, 0.051, 0.054, 0.41, and 0.65 mg/mL, respectively (Table 1). Furthermore, BBR-BSA NPs showed potent total antioxidant capacities (TAC) compared to the BBR, BSA, and BSA NPs and demonstrated concentration-dependent activities with EC_50_ values of 1.18 and 1.19 mg/mL for FRP and phosphomolybdenum complex, respectively (Table 1 and Supplementary Table S4).

**Table 1:** The inhibition or the effective concentrations (IC_50_ or EC_50_) of free radicals scavenging activities, total antioxidant capacities, and anti-hemolytic properties of the tested compounds.

Antioxidant potentials	Formulations				
	Inhibition or effective concentrations (IC50 or EC50) (mg/mL)				
	BBR	BBR-BSA NPs	Ascorbic acid	BSA	BSA NPs
NO^·^ radical scavenging	1.64 ± 0.35^a^	0.71 ± 0.11^b^	1.16 ± 0.28^c^	121.51 ± 2.88^d^	111.87 ± 2.29^e^
H_2_O_2_ radical scavenging	1.87 ± 0.23^a^	0.64 ± 0.16^b^	2.76 ± 0.33^c^	276.23 ± 3.44^d^	104.77 ± 3.11^e^
O_2_^−^ radical scavenging	3.62 ± 0.23^a^	1.21 ± 0.11^b^	5.11 ± 0.26^c^	176.21 ± 2.56^d^	100.95 ± 2.33^e^
OH– radical scavenging	0.31 ± 0.005^a^	0.051 ± 0.002^b^	1.02 ± 0.004^c^	75.11 ± 2.38^d^	48.83 ± 1.23^e^
DPPH radical scavenging	0.21 ± 0.004^a^	0.054 ± 0.002^b^	0.89 ± 0.017^c^	69.06 ± 3.11^d^	54.59 ± 2.88^e^
Liver TBARS radical scavenging	0.79 ± 0.05^a^	0.41 ± 0.03^b^	0.54 ± 0.04^c^	74.61 ± 2.11^d^	50.68 ± 1.66^e^
Brain TBARS radical scavenging	0.95 ± 0.05^a^	0.65 ± 0.02^b^	1.24 ± 0.07^c^	80.16 ± 3.17^d^	57.83 ± 2.22^e^
TAC/FRP	1.75 ± 0.09^a^	1.18 ± 0.05^b^	0.74 ± 0.007^c^	312.5 ± 5.62^d^	100.05 ± 2.83^e^
TAC/Green Phosphomolybdenum	1.55 ± 0.16^a^	1.19 ± 0.11^b^	0.59 ± 0.04^c^	218.22 ± 3.44^d^	115.11 ± 2.62^e^
RBCs lysis	42.11 ± 1.22^a^	229.64 ± 5.67^b^	40.62 ± 0.88^a^	504.81 ± 11.88^c^	1683 ± 34.77^d^

NO^·^, nitric oxide; H_2_O_2_, hydrogen peroxide; O_2_^−^, superoxide anion; OH^−^, hydroxyl radical; DPPH, 1, 1-diphenyl-2-picrylhydrazyl; TBARS, thiobarbituric acid reactive substances; TAC, Total antioxidant capacities; FRP, ferric reducing power; RBCs, red blood cells. Data values are expressed as means ± SD (n = 5). Different letters in the same row indicated statistically significant differences (*P* < 0.05) that evaluated using the analysis of one-way ANOVA with post hoc LSD test. The same letters in the same row indicated non-significant differences (*p* > 0.05).

Moreover, BSA and BSA NPs had the lowest antioxidant properties, including an inhibition rate of 21.33 and 26.33% against NO radical scavenging; 12.88 and 22.95% against H_2_O_2_ radical scavenging; 13.11 and 20.63% against O_2_^−^ radical scavenging; 28.11 and 41.22% against OH^−^ radical scavenging; 31.11 and 39.65% against DPPH radical scavenging; 28.11 and 39.71% against liver TBARS and 25.11 and 34.77% against brain TBARS at a concentration of 40 mg/mL. In addition, BSA and BSA NPs had higher IC_50_ values and lower antioxidant capacities (FRP and phosphomolybdenum complex) compared to BBR and BBR-BSA NPs, as illustrated in Table 1 and Supplementary Table S4. Interestingly, the antioxidant potential of BSA NPs was significantly (*P* < 0.05) increased compared to BSA, and BBR-BSA NPs also demonstrated a significant (*P* < 0.05) scavenging efficacy toward these free radicals compared to ascorbic acid (positive control) (Table 1 and Supplementary Table S4).

#### Estimation of the anti-hemolytic and anti-clotting properties of BBR-BSA NPs and BSA NPs

Supplementary Fig. S10 and Table S4 demonstrate that the tested compounds had potent anti-hemolytic activities within less than 5% hemolysis. Furthermore, BBR-BSA NPs had the lowest cytotoxic effect (*P* < 0.05) against the erythrocyte membrane integrity, as indicated by 0.47 ± 0.011% lysis compared to 3.11 ± 0.144% and 2.79 ± 0.163% of BBR and ascorbic acid, respectively, at the same concentration of 1.8 mg/mL. Furthermore, the IC_50_ values were 229.64 ± 5.67, 42.11 ± 1.22, and 40.62 ± 0.88 mg/mL for BBR-BSA NPs, BBR, and ascorbic acid, respectively. BSA NPs demonstrated significantly elevated anti-hemolytic powerful activity compared to BSA after formulation (*P* < 0.05). Conversely, BSA NPs have 1.29 ± 0.088% lysis and IC50 value of 1683 ± 34.77 mg/mL compared to that of BSA (4.37 ± 0.211% lysis and IC50 of 504.81 ± 11.88 mg/mL), Table 1 and Supplementary Table S4.

Concerning the anticoagulant activity, all the tested compounds significantly (*P* < 0.05 vs. negative control) increased the clotting time in a concentration-dependent time (Supplementary Fig. S11 and Table S4). Supplementary Table S4 illustrates that BBR-BSA NPs had the highest anticoagulant activity and activated partial thromboplastin time (APTT) of 92.7 ± 0.8 s compared to the coagulation time of BBR (79.4 ± 0.9 s), ascorbic acid (82.9 ± 1.1 s) at the same concentration of 1.8 mg/mL. In contrast, BSA and BSA NPs had lower APTT values. After formulation, BSA NPs significantly (*P* < 0.05) enhanced the anti-platelet coagulation activity and elevated the APPT value (67.7 ± 1.2 s) compared to BSA (58.8 ± 0.6 s) at 40 mg/mL (Supplementary Table S4).

#### Antimicrobial activities of BBR and BBR-BSA NPs

BSA and BSA NPs usage (0.5–40 BSA mg/mL) did not induce any zones of inhibition (mm) against *E. coli, C. albicans, B. subtilis, and S. aureus* pathogens (Supplementary Table S5)*.* The encapsulated BBR (0.01–1.8 BBR mg/mL) in the BSA NPs core substantially improved its antimicrobial activities against these pathogens compared to BBR (Supplementary Table S6). As illustrated in Table 2, BBR-BSA NPs demonstrated a significant (*P* < 0.05) increase in their inhibition zones (mm), with significantly decreased inhibitory concentrations (MICs) towards this microbial growth compared to BBR. In contrast, *E. col*i illustrated an inhibition zone of 32.7 ± 1.3 vs. 29.3 ± 1.5 mm and MICs of 6.1 ± 0.2 vs. 8.2 ± 0.2 μg/mL. *C. albicans* demonstrated an inhibition zone of 31.3 1.7 vs. 27.8 1.4 mm and MICs of 2.3 0.3 vs. 2.9 0.1 g/mL, whereas B. subtilis demonstrated an inhibition zone of 30.8 2.2 vs. 26.6 1.2 mm and MICs of 5.1 0.4 vs. 11.7 0.6 g.

**Table 2:** Antimicrobial activities of BBR and BBR-BSA NPs.

	*E. coli*	*C. albicans*	*B. subtilis*	*S. aureus*
**BBR**				
Zones of inhibition (mm)	29.3 ± 1.5^a^	27.8 ± 1.4^a^	26.6 ± 1.2^a^	30.8 ± 1.7
MIC (μg/mL)	8.2 ± 0.2^a^	2.9 ± 0.1^a^	11.7 ± 0.6^a^	10.8 ± 0.4^a^
**BBR-BSA NPs**				
Zones of inhibition (mm)	32.7 ± 1.3	31.3 ± 1.7	30.8 ± 2.2	32.7 ± 1.3
MIC (μg/mL)	6.1 ± 0.2	2.3 ± 0.3	5.1 ± 0.4	3.2 ± 0.1

Zones of inhibition (mm) were observed at a concentration of 1.8 mg BBR/mL. Minimum inhibitory concentrations (MICs) were introduced at μg/mL. Data values are expressed as means ± SD (n = 5).

^a^Represent a significant difference at *P* < 0.05 compared to BBR-BSA NPs as determined by the one-way ANOVA with post hoc LSD test.

### In vivo studies

#### The effect of BBR and BBR-BSA NPs on serum glycemic markers and pancreatic oxidative stress biomarkers

**Figure 6:**
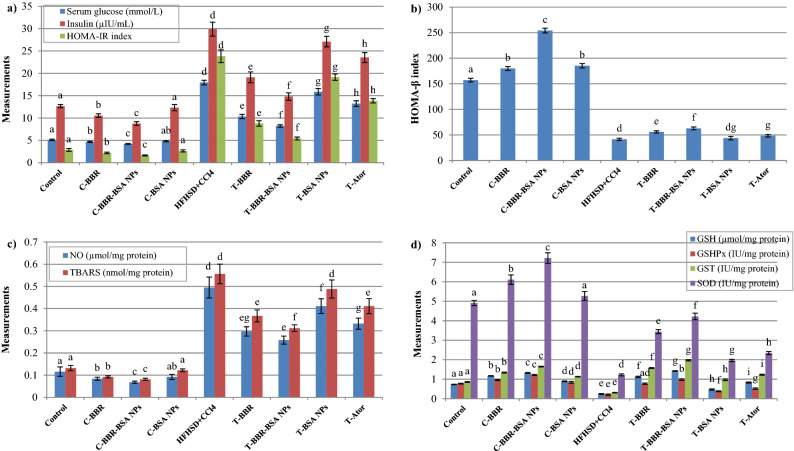
The effect of BBR and BBR-BSA NPs on the stressed pancreatic rat model. Serum glucose, insulin, and HOMA-IR index levels (**a**); Serum HOMA-β index (**b**); Pancreatic NO and TBARS levels (**c**); Pancreatic GSH level, GSHPx, GST, and SOD activities (**d**). Data values are expressed as means ± SD (n = 5). Different letters indicated statistically significant differences (*P* < 0.05) between the experimental groups within the same parameter, which were evaluated using the analysis of one-way ANOVA with the post hoc LSD test.

In the current study, the control (C)-BBR-BSA NPs group demonstrated a significant (*P* < 0.05) improvement in glucose metabolism and insulin sensitivity compared to the control rats, C-BBR, and C-BSA NPs groups. The stressed pancreatic rat model demonstrated a significant (*P* < 0.05) increase in the levels of serum glucose, insulin, and HOMA-IR index associated with a significant (*P* < 0.05) decrease in the level of HOMA-β index compared to the control group. The BBR-BSA NPs-treated rats (T-BBR-BSA NPs) significantly (*P* < 0.05) reduced the hyperglycemia and the peripheral insulin resistance features and elevated the insulin sensitivity and β-cell function (HOMA-β) compared to the stressed rats, BBR-, BSA NPs-, and Ator- treated rats (Fig. 6a,b).

In this study, the C-BBR-BSA NPs group demonstrated significantly decreased (*P* < 0.05) levels of pancreatic TBARS and NO and substantially increased (*P* < 0.05) the level of reduced pancreatic GSH as well as GSHPx, GST, and SOD activities compared to the control, C-BBR and C-BSA NPs groups. The administration of HFHSD plus carbon tetrachloride (CCl_4_) injections significantly (*P* < 0.05) increased the levels of pancreatic lipotoxicity and free radicals' generation (elevated TBARS and NO levels) and significantly (*P* < 0.05) decreased the antioxidant modulators (reduced GSH level as well as GSHPx, GST, and SOD activities). On the contrary, the BBR-BSA NPs-treated rats demonstrated a significant (*P* < 0.05) retardation in pancreatic toxicity and improved the pancreatic antioxidant biomarkers compared to the stressed, T-BBR, T-BSA NPs, and T-Ator groups (Fig. 6c,d).

#### The effect of BBR and BBR-BSA NPs on pancreatic histopathological evaluations

**Figure 7:**
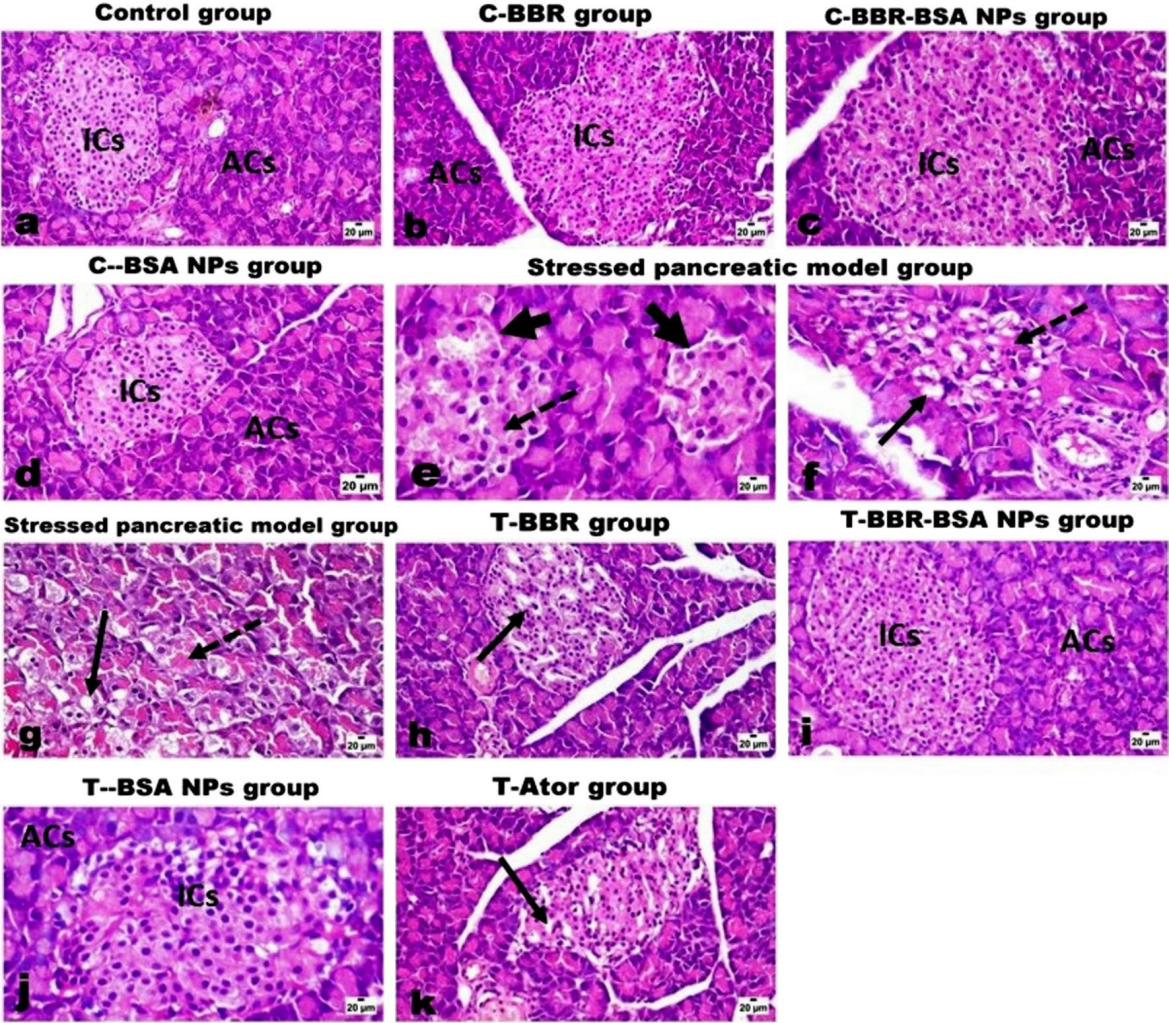
The histopathological investigations of the effect of BBR and BBR-BSA NPs on the stressed pancreatic rat model. Control (**a**), C-BBR (**b**), C-BBR-BSA NPs (**c**), and C-BSA NPs groups (**d**) show normal endocrine islets of Langerhans (ICs) and exocrine acinar cells (ACs). The stressed pancreatic rat model (**e**–**g**) shows a decrease in size and number of islets cells (thick arrow) with its necrosis (dashed arrow) (**e**), vacuolar degeneration (arrow), and necrosis (dashed arrow) of some islet cells (**f**), and marked swelling, vacuolation (arrow) and necrosis (dashed arrow) of pancreatic acinar cells (**g**). T-BBR group (**h**) shows scattered necrotic islets cells (arrow) and mild pancreatic congested vessels. T-BBR-BSA NPs group (**i**) shows normal islets cells (ICs) as well as the acinar cells (ACs) with only a few islets' cells with pyknotic nuclei. T-BSA NPs group (**j**) shows normal ACs and mild cytoplasmic vacuolation of a few islets' cells. T-Ator group (**k**) shows a mild degree of islets cell degeneration (arrow) and scattered necrosis. (H&E, X200, scale bar = 20 μm).

The histopathological investigations revealed that control, C-BBR, C-BBR-BSA NPs, and C-BSA NPs groups had normal pancreatic endocrine parts (islets of Langerhans) and healthy exocrine acinar cells (ACs) (Fig. 7a–d). While the pancreatic tissue of stress- rats model (HFHSD administration plus CCl_4_ injection) revealed marked histological alterations in both endocrine and exocrine parts (Fig. 7e–g). The Islets of Langerhans appeared smaller in size and lower in number compared to the control islets (Fig. 7e) and showed vacuolar degeneration and necrosis (Fig. 7f). Moreover, the necrotic cells appeared either with pyknotic nuclei or homogenous eosinophilic structureless without nuclear structure. The pancreatic duct also showed mild to a moderate proliferation of its epithelial linings (Fig. 7e,f). Figure 7g demonstrates that the stressed pancreatic rat model demonstrated severe swelling of the acinar cells with marked vacuolar degeneration and loss of zymogen granules.

Regarding the treated groups, BBR- the treated group (T-BBR) showed a moderate curative effect on the pancreatic tissue with some degenerated and scattered necrotic islets cells and mild pancreatic congested vessels (Fig. 7h). BBR-BSA NPs treatment was described as a good restorative formulation that significantly improved the pancreatic exocrine and endocrine sections and showed near to normal appearance of both islets cells as well as the acinar cells (Fig. 7i). The BSA NPs-treated rats also demonstrated normal pancreatic tissue with mild cytoplasmic vacuolation of few islets’ cells (Fig. 7j). In addition, the Ator- treated group demonstrated a mild degree of islet cells degeneration, scattered necrosis, and few pyknotic cells (Fig. 7k). Interestingly, treatment with BBR-BSA NPs had a more restorative effect on pancreatic tissue than the treatment with BBR followed by the treatment with reference drug (Ator).

## Discussion

Bovine serum albumin, a nano-carrier, can encapsulate several bioactive components and deliver different drugs and natural products with high selectivity, high bioavailability, and potent therapeutic properties^23,26,27^. In the current study, BBR-BSA NPs were synthesized by the desolvation method, using ethanol as a desolvating agent and glutaraldehyde as the crosslinking agent. The PDI value indicates the homogeneity of the prepared NPs. The lower PDI value revealed a better uniform distribution of the prepared NPs, demonstrating good stability^28^. In the present study, the synthesized alkaline BBR-BSA NPs have better properties than the neutral ones as the particle size diameter, and PDI values decreased, and their zeta potential increased. Previous studies reported that shifting away from the isoelectric point of BSA by increasing the pH of the NPs preparation media reduced the coagulation interactions and induced the electrostatic repulsive forces between the BSA molecules^27,29,30^. Moreover, the surface charge of the formulation is considered an indicator of the stability of the NPs distribution, which indicates the degree of electrostatic repulsion between the particles^28^. The highly charged particles have a high zeta potential value, considered stable due to the strong electric repulsion forces between the molecules^28^. NPs with a particle size < 200 nm can significantly avoid renal clearance and permeate into different tissues^28^. In this study, the alkaline BBR-BSA NPs size diameter was < 200 nm. Interestingly, the hydrodynamic size diameter of the alkaline BBR-BSA NPs (155 ± 0.6 nm, hydrated form) was relatively higher than their diameter from FE-TEM measurements (125 ± 23.60 nm, dried form). During DLS measurements, the presence of water around BBR-BSA NPs increased their size diameter compared to the TEM measurements^14,15^.

In agreement with Stoyanova et al*.* report., our results confirmed that the entrapment of BBR in the BSA NPs core increased its thermal stability by decreasing the values of the mass-loss rate, heat change, and enthalpy of BBR related to the increased temperature^31^. In addition, the retardation in the mass degradation rates and the intensities of the melting peaks of the individual target compounds related to the increased temperature indicated the transformation from their physical crystalline states to the NPs' amorphous form^31^. Therefore, the nano-particulate drug delivery system is introduced as a vital tool to increase the thermal stability of the entrapped bioactive compounds^31^.

BSA NPs could increase the half-life time, LC%, and the antioxidant properties of the entrapped bioactive ingredients^24^. The solubility of BBR-BSA NPs formed in distilled water, 0.1 M HCl, and PBS (pH 6.8) was significantly increased compared to BBR^24^. After formulation, the synthesized BSA NPs entrapped BBR in an amorphous state, reducing the agglomeration and aggregation between the hydrophobic BBR particles^24^. Our study targeted the encapsulation of standard BBR-chloride in the core of BSA NPs that delayed and minimized the BBR releasing rates in different dialysis media (SIF or SGF), which agrees with Patra et al*.*^32^ and Saleh et al*.*^33^. Wu et al*.* also reported that chitosan/fucoidan-taurine conjugate NPs released BBR in SIF (pH 7.4) faster than its releasing rate in SGF (pH 2.0)^15^.

Furthermore, the current in vitro results revealed that BBR-BSA NPs greatly enhanced the antioxidant properties of its entrapped BBR against NO^·^, H_2_O_2_, O_2_^−^, OH^−^, DPPH, TBARS, and potassium ferricyanide/ferric chloride, and ammonium molybdate radicals compared to BBR. BBR-loaded polymeric nanostructures have been used to improve the physicochemical characteristics (solubility, dissolution rate, hydrophilicity, and releasing profile), pharmacodynamics and pharmacokinetics properties, selectivity, and the therapeutic potentials of BBR^28,34^. BBR was reported as a potential anti-radical alkaloid that scavenges several free radicals such as DPPH, NO^·^, H_2_O_2_, O_2_^−^, lipid peroxidation, and ferric ions^35,36^ and BBR NPs formulations were found to increase the radical scavenging activity^31^. In the current study, BSA NPs as a nano-carrier markedly elevated the anti-hemolytic potent activity and reduced the cytotoxicity of the entrapped BBR. Previous work confirmed that BSA NPs could improve the therapeutic properties and attenuate the toxicity of several bioactive compounds^22^. By augmenting the anticoagulant activity of BBR, our findings suggested that BSA nanoparticles could significantly stimulate and improve blood flow .

BBR has potent antimicrobial properties against bacteria, viruses, protozoa, fungi, and yeasts^37^, which supports its antibiotic action toward harmful pathogenic bacteria^38,39^. Previous studies aimed to develop a natural antimicrobial nano-particulate drug delivery formulation that could enhance the sensitivity of the antimicrobial drugs^40,41^. Our study demonstrated that BBR-BSA NPs demonstrated the highest antimicrobial activities and the lowest MIC values compared to BBR against the studied pathogenic microbes, which confirmed the results of the Sahibzada et al*.* work^42^. Moreover, BBR-conjugated chitosan or alginate nanostructures revealed more potent antibacterial activities compared to BBR hydrochloride^34^.

Excessive consumption of high fat/ high sugar diets mainly develops hepatic steatosis, oxidant generation, chronic liver injury, necro-inflammation, and mitochondrial dysfunction^43–45^. CCl_4_, a strong hepatotoxin, is metabolized to produce a highly reactive substance known as trichloromethyl radical (CCl_3_^*^). This radical can bind with cellular molecules to generate oxidants and cellular damage and disturb mitochondrial homeostasis^46,47^. CCl_3_^*^ can react with oxygen to form highly reactive species that initiate the chain reaction of lipid peroxidation^46,47^. Furthermore, HFHSD feeding plus streptozotocin (STZ) injection also developed a diabetic mice model that described hyperglycemia and hyperinsulinemia and a severe injury in the islets of Langerhans (β-cells) and pancreatic ACs^48^. Furthermore, previous studies reported that exposure to HFD and/or CCl_4_ developed severe degeneration and necrosis in the pancreatic tissues and inhibited pancreatic β-cells survival^49–51^. These findings agree with the current results where HFHSD/ CCl_4_ stress model induced pancreatic oxidative stress, hyperglycemia, and hyperinsulinemia and demonstrated significant histological alterations in the pancreatic endocrine parts (islets of Langerhans) and the exocrine acinar cells. These findings indicated a reduction in the function and sensitivity of β-cells islet upon HFHSD/CCl_4_ stress^52,53^.

The current in vivo studies evaluated the enhanced protective effect of BBR-BSA NPs on HFHSD/ CCl_4_ stressed pancreatic tissue. Natural products have an essential role as insulin sensitizers. BBR, as an alkaloid compound, is a potent hypoglycemic modulator, significantly improved the function of pancreatic β-cells, restored glucose homeostasis, and enhanced the peripheral insulin sensitivity^54^ of HF fed^55^, HFHSD fed^53^, and STZ-induced diabetic rats^2^. In the current study, BBR administration was found to improve the level of the glycemic markers (serum glucose, insulin, as well as HOMA-IR and HOMA-β index) and the level of the pancreatic oxidative stress parameters (TBARS, NO, GSH level, and GSHPx, GST and SOD activities). BBR-BSA NPs administration demonstrated powerful therapeutic antioxidant properties, which substantially improved the pancreatic injury features and restored pancreatic homeostasis compared to the other treatments under control and stress conditions. In addition, BBR-BSA NPs administration has therapeutic efficacy against the induced pancreatic injury and exhibited a significant curative effect on the histopathological alterations of pancreatic areas and the function of pancreatic ACs and β-cells. These findings are compatible with previous studies conducted on BBR^2,53,54,56^.

### Conclusion

According to physicochemical characteristics and in vitro and in vivo studies of BBR-BSA NPs, BBR-BSA NPs delayed the mass degradation rate and increased thermal stability of the entrapped BBR compared to BBR-chloride. Furthermore, BBR-BSA NPs successfully reduced in vitro BBR releasing rates in different dialysis media (SIF and SGF), which decreased its local dissolution rates compared to BBR-chloride. BBR-BSA NPs demonstrated potent free radical scavenging activities, total antioxidant capacities, anti-hemolytic and anticoagulant properties, and antimicrobial potentials compared to BBR-chloride. In addition, BBR-BSA NPs significantly reduced the levels of pancreatic injury and oxidants generation and improved the glucose homeostasis, and insulin sensitivity of the HFHSD/CCl_4_-induced stressed pancreatic rat model compared to BBR-chloride (Supplementary Fig. S12a-d).

## Materials and methods

### Materials

Berberine chloride [BBR-chloride (C_20_H_18_ClNO_4_), natural yellow 18, Mw 371.81 g/mole, B3251-25G] and glutaraldehyde (25%, v/v crosslinking agent) were purchased from Sigma-Aldrich (St. Louis, MO, USA). Analytical HPLC-grade ethanol (desolvating agent) was purchased from Fisher Chemicals (USA). Ultrapure water (deionized water) is produced by the Milli-Q synthesis system (Millipore Corp., Billerica, MA, USA). Bovine serum albumin (BSA) lyophilized form (P6154-10GR, pH ~ 7, Mw 66 KDa) supplied from Biowest (France). The activated partial thromboplastin time (APTT) kit was purchased from Biomed Diagnostics Co., Ltd. (Egypt). Other chemicals and reagents were an analytical grade. In this study, all solutions and buffers were prepared in ultrapure deionized water.

### Preparation and physicochemical characterization of BBR-loaded albumin nanoparticles

#### Preparation of BBR-BSA NPs and BSA NPs

BBR-BSA NPs and BSA NPs were prepared by the desolvation method with specific modifications^21,23,27,30^. As described in Supplementary Table S1, 200 mg of BSA was dissolved in 5 mL of either deionized water (pH 7.2) or deionized water under alkaline conditions (0.1 M NaOH, pH 10). The solution was stirred at 750 rpm for 30 min at 25 °C. In order to form BBR-BSA NPs, 20 mg of BBR was incubated within the polymeric solution for 2 h. The NPs were produced by the drop-wise addition of a specific volume of ethanol at a rate of 1 mL/min to the BBR-BSA complex solution under continuous magnetic stirring (750 rpm) until the solution became turbid.

Afterward, the prepared BBR-BSA NPs were crosslinked with a specific volume of 8% glutaraldehyde. The suspended NPs were stirred until all ethanol and glutaraldehyde were evaporated. The resulting NPs pellet was purified using differential centrifugation at 14,000 rpm through 6 cycles (each cycle 20 min) at 4 °C until the supernatant became transparent. The NPs pellet was washed with deionized water to remove the free drug, BSA, and glutaraldehyde molecules from the surface of the NPs. The NPs pellet was also suspended and redispersed to reach the original volume (5 mL) with deionized water as a vehicle using a stick-type ultrasonicator^15^. Specific formulations were stalled as suspended forms, and others were lyophilized at − 48 °C for 24 h, weighed, and stored in the sealed vials at 4 °C. The pH was adjusted with 0.1 M NaOH solution through the NPs preparation step (Supplementary Table S1).

#### Determination of the mean particle diameter, particle size distribution (polydispersity indices), zeta potential, and surface morphology of the synthesized NPs

The hydrodynamic particle size diameter, polydispersity indices (PDI), and zeta potential of the synthesized NPs were measured by Dynamic Light Scattering (DLS)/photon correlation spectroscopy using a particle seizer at a fixed angle of 90° at 25 °C [Zetasizer Ver. 6.20 (Serial Number: MAL1054905), Malvern Instruments Ltd., UK]. Furthermore, field emission-transmission electron microscopy (FE-TEM) photomicrographs were used to evaluate the surface morphological characterization and the size of BBR-BSA NPs and BSA NPs. For analysis, the TEM samples were measured by JSM 1400 PLUS-JEOL. To perform the FE-TEM observations, the suspended NPs were first diluted with deionized water (40 µL of suspended NPs was added to 15 mL of deionized water). Then, a drop of the diluted suspended NPs was placed onto a copper grid coated with carbon film. The excess was drawn off with a dry filter paper. The copper grid was allowed to dry at room temperature before its scanning at various magnifications.

#### Ultraviolet–Visible spectroscopic measurements

The unloaded BBR and BSA contents (mg) were determined by measuring the free BBR and BSA concentrations in the supernatant using an Ultraviolet–Visible (UV–Vis) spectrophotometer. Moreover, the BBR encapsulation efficiency (EE%), its loading capacity (LC%), and the NPs synthesis yield (SY%) were calculated by the following equations:

$${\text{EE}}\left( \% \right) = \left[ {{\text{Initial}}\;{\text{BBR}}\left( {{\text{mg}}} \right){-}{\text{Free}}\;{\text{BBR}}\;{\text{in}}\;{\text{supernatant}}\left( {{\text{mg}}} \right)/{\text{Initial}}\;{\text{BBR}}\left( {{\text{mg}}} \right)} \right]{*1}00$$

$${\text{LC}}\left( \% \right) = \left[ {{\text{Initial}}\;{\text{BBR}}\left( {{\text{mg}}} \right){-}{\text{Free}}\;{\text{BBR}}\;{\text{in}}\;{\text{supernatant}}\left( {{\text{mg}}} \right)/{\text{NPs}}\;{\text{lyophilized}}\;{\text{form}}\left( {{\text{mg}}} \right)} \right]*{1}00$$

$${\text{SY}}\left( \% \right) = \left[ {{\text{NPs}}\;{\text{lyophilized}}\;{\text{form}}\left( {{\text{mg}}} \right)/\left( {{\text{Initial}}\;{\text{BBR}}\left( {{\text{mg}}} \right) + {\text{Initial}}\;{\text{BSA}}\left( {{\text{mg}}} \right)} \right)} \right]*{1}00$$

#### Fourier transforms infrared spectroscopic analysis

Fourier transforms infrared (FTIR) spectroscopy is an absorption spectrophotometer that can identify the different chemical compounds depending on their chemical structures and functional groups^57^. The FTIR samples were prepared by grinding 98.99% KBr with 1% of the samples that were compressed to form a tablet^26^. Pure BBR-chloride, pure BSA, BBR-BSA NPs, and BSA NPs were characterized using an FTIR spectrometer (Bruker Tensor 37 FTIR spectroscopy with ATR method, German).

#### Thermal stability of the synthesized nanoparticles

The thermodynamic behavior, decomposition rates with increasing temperature, thermal stability, and the variations in the loss rates of the amorphous matter (BBR-BSA NPs and BSA NPs) and its crystalline form (BBR and BSA) were carried out using LINSEIS STA PT 1000 (TG–DTA/DSC). The temperature of this program began from 0 to 700 ℃, with a heating rate of 10 ℃/min. Furthermore, the thermal gravimetric analysis (TGA) profile showed the temperature stability and the variations in the mass change rates of specific compounds (crystalline forms or amorphous states) related to the increased temperature^58^. Differential thermal analysis (DTA) and differential scanning calorimetry (DSC) profiles were also used to investigate the existence of the drug in the NPs form^16,59^.

### *In vitro* studies

#### BBR releasing profile

In this study, the releasing profile of BBR from BBR-BSA NPs (pH and time-dependent stability) was estimated using a dialysis tubing (Nadir®-Dialysierschläuche, 38 mm, 25–30 Å, MWCO ca. 10,000–20,000 Dalton) in different dialysis media. A 0.1 M phosphate-buffered saline (PBS, pH 7.4) and 0.01 N HCl (pH 2.0) were introduced as dialysis media at 37.0 ± 0.5 °C and 75 rpm using a Thermo-Shaker (Thermo Scientific, USA, model. 4352). These media were considered simulated intestinal fluid (SIF) and gastric fluid (SGF), respectively, to simulate the physiological conditions following oral administration^15,33,60^.

In 100 mL beaker, the dialysis bag which contained 5 mg of BBR or BBR-BSA NPs was immersed in 50 mL of different dialysis media (SIF or SGF). Aliquots of the dialysis media that contained released BBR were withdrawn at time intervals (0, 0.5, 1, 2, 3, 4, 6, 8, 12, 16, 20, 24, 28, 32, 36, 40, 48 h.). In order to maintain the releasing media, the media was completed to 50 mL after each withdrawn step. The concentration of the released BBR was evaluated by determining the absorbance of BBR at 344 nm using a UV–Vis spectrophotometer. The cumulative BBR releasing rate (%) in the dialysis medium was calculated according to Wu, et al.^15^ as follows:

1 $${\text{Cumulative}}\;{\text{BBR}}\;{\text{releasing}}\;{\text{rate}}\left( \% \right) = \left[ {{\text{Released}}\;{\text{BBR}}\left( {{\text{mg}}} \right)/{\text{Initial}}\;{\text{BBR}}\left( {{\text{mg}}} \right)} \right]*{1}00$$

#### Determination of the nitric oxide (NO^·^), hydrogen peroxide (H_2_O_2_), superoxide anion (O_2_^−^), and hydroxyl radicals (OH^−^) scavenging activities

NO^**·**^ scavenging activities (%) of standard BBR-chloride (0.01–1.8 mg/mL), BBR-BSA NPs (0.01–1.8 mg BBR/mL), BSA (0.5–40 mg BSA/mL) and BSA NPs (0.5–40 mg BSA NPs/mL) in addition to ascorbic acid (0.01–1.8 mg/mL, reference standard) and sodium nitrite (NaNO_2_, 5–232 µM, as positive controls), were determined using the Griess reaction method with minor modifications^58^. Furthermore, hydrogen peroxide (H_2_O_2_), superoxide anion (O_2_^−^), and hydroxyl radicals (OH−) scavenging activities of tested compounds were determined according to standard methods with slight modifications^36,61,62^. The modified procedures used to determine the free radical scavenging activities of these compounds are explained in detail in our supplementary file. The percentage of the scavenged free radicals was estimated as follows:

2 $${\text{Free}}\;{\text{radicals}}\;{\text{scavenging}}\;{\text{activity}}\left( \% \right){ = }\left[ {\left( {{\text{Ac}} - {\text{At}}} \right){\text{/Ac}}} \right]{*100}$$

Ac, the absorbance of the negative control against its blank; At, the absorbance of the different diluted samples and the reference standard (positive control) against their blank. The value of the inhibitory concentration 50 (IC_50_) represented the sample concentration (mg/mL) that inhibited 50% of the oxidant agent (radical) activity.

#### Determination of the 1, 1-diphenyl-2-picrylhydrazyl (DPPH) scavenging activity

The dark purple DPPH gradually turns to a clear yellow color when it is reduced by an antioxidant agent. These samples' DPPH radical scavenging activities were evaluated using the standard method with minor modifications (Supplementary file)^1,58^. The percentage of the scavenged DPPH was determined as the following:

3 $${\text{DPPH}}\;{\text{scavenging}}\;{\text{activity}}\left( \% \right) = \left[ {\left( {{\text{Ac}} - {\text{At}}} \right)/{\text{Ac}}} \right]*100$$

#### Determination of the thiobarbituric acid reactive substances (TBARS) scavenging activity

TBARS scavenging activities (%) of BBR, BBR-BSA NPs, BSA, BSA NPs, and ascorbic acid were determined according to the standard method^62,63^ with some modifications explained in detail in the supplementary file. The TBARS scavenging activities (%) were evaluated as the following:

4 $${\text{TBARS}}\;{\text{scavenging}}\;{\text{activity}}\left( \% \right) = \left[ {\left( {{\text{Ac}} - {\text{At}}} \right)/{\text{Ac}}} \right]*100$$

Ac, the absorbance of the negative control; At, the absorbance of the different diluted samples and the reference standard (positive control) against their blank.

#### Determination of the total antioxidant capacities (TAC)

The total antioxidant capacities (TAC) of tested compounds and corresponded NPs besides ascorbic acid (reference drug) were determined using the potassium ferricyanide/ferric chloride reducing power (FRP) and green phosphomolybdenum complex methods with slight modifications (Supplementary file)^58,62^. The effective concentration value 50 (EC50) represented the compound concentrations (mg/mL) that had 50% antioxidant reducing power.

#### Determination of the anti-hemolytic powerful and the anticoagulant activity

The effect of BBR, BBR-BSA NPs, BSA, BSA NPs, and ascorbic acid on the red blood cells lysis rate (%)was determined with minor modifications mentioned in the supplementary file^24,64^. After the incubation period with the prepared compounds, the released hemoglobin was quantified to measure the red blood cells lysis rate (%), which normalized to the 100% released hemoglobin of the positive control (Triton X-100). The hemolysis (%) was estimated as follows:

5 $${\text{Hemolysis}}\left( \% \right) = \left[ {\left( {{\text{A}}_{{{\text{sample}}}} - {\text{A}}_{{{\text{C}} - }} } \right)/\left( {{\text{A}}_{{{\text{C}} + }} - {\text{A}}_{{{\text{C}} - }} } \right)} \right]*{1}00$$

where A _sample_ was the absorbance of the different diluted tested samples; A_C+_ was the absorbance of the positive control (100% hemolysis); A_C-_ was the absorbance of the Mcllvaine's buffer (blank) or the diluted erythrocytes suspended form (4% hematocrit) in the Mcllvaine's buffer (negative control). The value of the inhibition concentration (IC_50_) was introduced as the compound concentrations (mg/mL) that had 50% anti-hemolytic power.

Concerning the anticoagulant activity, BBR (0.01–1.8 mg/mL), BBR-BSA NPs (0.01–1.8 mg BBR/mL), BSA (0.5–40 mg BSA/mL), BSA NPs (0.5–40 mg BSA NPs/mL) and ascorbic acid (0.01–1.8 mg/mL, reference standard) were incubated with human plasma then activated partial thromboplastin time (APTT) was measured in order to determine their anticoagulant effects^65,66^.

#### Antimicrobial activity assay

The effect of BBR (0.01–1.8 mg/mL), BBR-BSA NPs (0.01–1.8 mg BBR/mL), BSA (0.5–40 mg BSA/mL) and BSA NPs (0.5–40 mg BSA NPs/mL) on the inhibition growth rate of different pathogenic microbes was detected using Luria–Bertani (LB) agar diffusion well-variant method^67^. Where, Gram-positive bacteria as *Bacillus subtilis* (*B. subtilis*) and *Staphylococcus aureus (S. aureus*) and Gram-negative bacteria as *Escherichia coli* (*E. coli*, ATCC 11,775)*; Candida albicans* (*C. albicans*, DSM 70,014) as yeast were used. The microbial inoculum was spread by a sterile cotton swab on a sterile petri dish LB agar media. Furthermore, 200 μL of different serial dilutions of BBR, BBR-BSA NPs, BSA, and BSA NPs were added to the wells of each pathogen. The plates were incubated for 24 h at 36 ± 1 °C under aerobic conditions (10% CO_2_ incubator). After incubation, the microbial growth inhibition zones were measured in mm. The minimum inhibitory concentration (MIC, μg/mL) was defined as the lowest concentration, with no visible microbial growth observed after 24 h of inoculation.

### In vivo studies

#### Experimental animals

Seventy-two male Wistar Albino rats (80–100 g) were purchased from the experimental animal house, Institute of Graduate Studies and Research (IGSR), Alexandria University, Alexandria, Egypt. The animals were kept in polypropylene cages covered with metallic grids and maintained at a proper environmental condition of a 12 h light/12 h dark cycle at 20–25 °C and 60 ± 5% relative humidity. Rats had to access the diet and drinking water ad libitum during the experimental period. All the experimental procedures were performed according to the criteria outlined in the Guide for the Care, and Use of Laboratory Animals, approved by the Institutional Animal Care and Use Committees (IACUCs) of Pharmaceutical and Fermentation Industries Development Center, Scientific Research and Technological Application City (Approval number: 31-1Z-1120) and the study was carried out in compliance with the ARRIVE guidelines.

The experimental work was designed to evaluate the harmful effects of the high-fat high sucrose diet (HFHSD) feeding an addition to carbon tetrachloride (CCl_4_) injection on the cellular homeostasis and the oxidative stress response of the pancreas. Furthermore, the standard control diet and HFHSD were prepared with minor modifications and fed along the experimental period of 15 weeks^48,68^. Moreover, CCl_4_, a cytotoxin, was also sub-cutaneously injected with slight modifications as a 50% CCl_4_ dosage with olive oil (3 ml/kg, twice weekly during the last three experimental weeks)^36,50,69^.

#### Animal design

After two weeks of acclimatization, the rats were categorized into nine groups (8 per each): the control (C) group received a standard control diet plus saline via intra-gastric gavage during the last four experimental weeks; the C-BBR, C-BBR-BSA NPs, and C-BSA NPs groups were orally received standard control diet plus BBR (150 mg BBR/kg/day, 6 days per week)^55^, BBR-BSA NPs (15 mg BBR/kg/day, 6 days per week), and BSA NPs (150 mg BSA NPs/kg/day, 6 days per week), respectively via intra-gastric gavage during the last four experimental weeks. The induced group received HFHSD feeding plus CCl_4_ injection. The T-BBR, T-BBR-BSA NPs, T-BSA NPs, and T-Ator groups were received HFHSD feeding plus CCl_4_ injection combined with the oral administration of BBR (150 mg BBR/kg/day, 6 days per week), BBR-BSA NPs (15 mg BBR/kg/day, 6 days per week), BSA NPs (150 mg BSA NPs/kg/day, 6 days per week), and Atorvastatin (10 mg Ator/kg/day, 6 days per week)^70^, respectively via intra-gastric gavage during the last four experimental weeks. Atorvastatin (Ator), a cholesterol-lowering agent, was used to upregulate the sensitivity and function of pancreatic β-cells of hyperglycemic and dyslipidemic animal models^71^.

At the end of the experiment, the rats fasted overnight and were sacrificed by decapitation under anesthesia (isofuran). Blood samples were collected by heart puncture and allowed for clotting at room temperature for 30 min. The serum samples were centrifuged at 4,000 rpm for 10 min at 4 °C and stored at −20 °C until the biochemical assays were done. The pancreas tissue was immediately removed and washed in ice-cold saline. For biochemical analysis, one gram of pancreas tissue was homogenized (10%, w/v, 1:9) in ice-cold 10 mM sodium–potassium phosphate buffer (pH 7.4) containing 1.15% potassium chloride (KCl). The tissue homogenate was centrifuged at 10,000 rpm for 20 min at 4 °C. The resulted supernatant was stored at – 80 °C to evaluate the oxidative stress biomarkers and antioxidant modulators' levels and activities. The pancreas tissue was fixed overnight in 10% neutral buffered formalin for histopathological investigations.

#### Routine biochemical measurements

The level of fasting serum glucose was determined by the colorimetric method using a VITRO SCIENT kit, Egypt, according to the kit instructions. Serum insulin concentration was measured by the quantitative sandwich enzyme immunoassay technique using an insulin quantitative immunoassay ELISA kit, USA, according to the manufacturer’s instructions. The homeostatic model assessment of insulin resistance (HOMA-IR) index was calculated as the following:

6 $${\text{HOMA - IR}}\;{\text{index}} = {\text{fasting}}\;{\text{serum}}\;{\text{glucose}}\;\left( {\text{mmol/L}} \right){\text{*fasting}}\;{\text{serum }}\;{\text{insulin}}\;\left( \mu {{\text{ IU/mL}}} \right){/22}{\text{.5}}$$

The homeostatic model assessment of the β-cell function (HOMA-β) index was evaluated as the following^52^:

7 $${\text{HOMA - }} \beta \;{\text{index}} = {20}\,{\text{*fasting}}\;{\text{serum}}\;{\text{insulin}}\;\left( \mu {{\text{ IU/mL}}} \right){\text{/fasting}}\;{\text{serum}}\;{\text{glucose}}\;\left( {\text{mmol/L}} \right) - {3}{\text{.5}}$$

The levels of pancreatic NO (μmol/mg protein), TBARS (nmol/mg protein), and reduced GSH (μmol/mg protein) were evaluated using methods that were determined previously by certain studies^72–74^. While the activities of pancreatic GSHPx (EC 1.11.1.9), GST (EC 2.5.1.18), and SOD (EC 1.15.1.1,) enzymes in IU/mg protein were estimated using methods that reported previously^75–77^.

#### Histopathological examinations

Pancreatic specimens were fixed in 10% neutral buffered formalin for histopathological analysis. After fixation, these tissues were processed and embedded in paraffin blocks. The solid sections were cut into a 5–6 µm thick, stained with hematoxylin and eosin (H&E) stain for histological evaluations. The pancreatic sections were examined under light microscopy (Olympus, Tokyo, Japan), and their photomicrographs were taken (X200 magnifications).

### Statistical analysis

For in vitro and in vivo studies, all data are statistically expressed as means ± standard deviations of the means (means ± SD). Statistical significance (*P* < 0.05) of differences were evaluated using the one-way ANOVA analysis of variance with a post hoc LSD test of SPSS Windows Version 19.0 (SPSS, Inc., Chicago, IL, USA) for multiple comparisons. The concentration of samples could be provided a 50% inhibition or effectiveness (IC_50_ or EC_50_), obtained by linear regression analysis. The ClustVis web server applications are freely available at http://biit.cs.ut.ee/clustvis/ that are introduced to summarize the data values in an ideal heatmap distribution analysis^78^.

## Supplementary Information

Supplementary Information.

## Data Availability

The datasets generated during the current study are available from the corresponding author on reasonable request.
