# Supplementary material for: Preparation, physicochemical characterization, and bioactivity evaluation of berberine-entrapped albumin nanoparticles
Source: Sci Rep. 2022 Oct 19;12:17431. doi: 10.1038/s41598-022-21568-8 (PMC9581884; doi:10.1038/s41598-022-21568-8)
Supplement: Supplementary file 1 — Supplementary Information. [file 41598_2022_21568_MOESM1_ESM.docx]

**Preparation, Physicochemical Characterization, and Bioactivity Evaluation of Berberine-Entrapped Albumin Nanoparticles**

Fatema A. Younis^1^, Samar R. Saleh^1*^, Sahar S. Abd El-Rahman^2^, Al-Sayeda A. Newairy^3^, Maha A. El-Demellawy^4,5^, and Doaa A. Ghareeb^1^

^1^ Bio-screening and Preclinical Trial Lab, Biochemistry Department, Faculty of Science, Alexandria University, Alexandria, Egypt.

^2^ Department of Pathology, Faculty of Veterinary Medicine, Cairo University, Giza, Egypt.

^3^ Biochemistry Department, Faculty of Science, Alexandria University, Alexandria, Egypt.

^4^ Pharmaceutical and Fermentation Industries Development Centre (PFIDC), the City of Scientific Research and Technological Applications (SRTA-City), Borg Al-Arab, Alexandria, Egypt.

^5^ Medical Biotechnology Department, GEBRI, SRTA-City, New Borg El-Arab City, Alexandria, Egypt.

***** Corresponding author: [samar.saleh@alexu.edu.eg](mailto:samar.saleh@alexu.edu.eg); ssaleh84@yahoo.com; Tel.: +20 1225732849.

# Preparation of BBR-BSA NPs and BSA NPs

Supplementary Table S1. Preparation of BSA NPs and BBR-BSA NPs using the desolvation method under neutral (deionized water) and alkaline conditions

|  | BBR  (mg) | BSA  (mg) | Solvent  (Deionized water,  mL) | 0.1M NaOH  (mL) | Ethanol  (mL) | 8% Glutaraldehyde  (μL) | PH |
| --- | --- | --- | --- | --- | --- | --- | --- |
| BSA NPs | 0 | 200 | 5 | …… | 8.2 | 15 | 7.2 |
|  |  |  |  | 0.5 | 16.2 |  | 10 |
| BBR-BSA NPs | 20 | 200 | 5 | …… | 6.55 | 59 | 7.2 |
|  |  |  |  | 0.5 | 14.2 | 35 | 10 |

# FTIR Spectroscopy:

After formulation, the FTIR spectrum of BBR-BSA NPs revealed the characteristic peaks of the NPs (Fig. 2c). The peak at 3381.15 cm^-1^ demonstrated the existence of the –OH stretching vibration band of BBR and BSA. The peak at 2926.32 cm^-1^ indicated the presence of the saturated C-H stretching band from BBR and the amide A band of BSA. The peak at 2860.43 cm^-1^ indicated the existence of the methoxyl group band from BBR and the amide B band of BSA. The peak at 1653.96 cm^-1^ denoted the presence of the heterocyclic amines band from BBR and the amide I band of BSA. The peak at 1538.46 cm^-1^ demonstrated the existence of the heterocyclic amines band, the C=C bending vibration band from BBR, and the amide II band of BSA. The peak at 1448.38 cm^-1^ demonstrated the presence of the C=C stretching vibration band, the furyl group band from BBR, and the CH_2_ bending group band of BSA. The peak at 1391.14 cm^-1^ demonstrated the presence of the –CH_3_ stretching vibrations band from BBR and BSA's CH2 bending group band. In contrast, the peak at 1236.77 cm^-1^ explained the presence of the C-O-C bonding band from BBR and the amide III band of BSA. Finally, the peak at 618.83 cm^-1^ exhibited the chloride ions band of the standard BBR-chloride (Supplementary Table **S2** and Supplementary Table **S3**).

The FTIR spectrum of BSA NPs explained the characteristic peaks of the NPs after formulation (Fig. 2d). The slightly shifting and/or changing in the intensities of these peaks resulted from the conformational changes between the BSA molecules after formulation. The peak at 3304.05 cm^-1^ demonstrated the existence of the –OH stretching vibrations band of BSA. The peak at 2926.09 cm^-1^ indicated the presence of the amide A band of BSA. The peak at 2857.37 cm^-1^ introduced the existence of the amide B band of BSA. The peak at 1655.34 cm^-1^ is related to the presence of the amide I band of BSA. The peak at 1538.62 cm^-1^ explained the existence of the amide II band of BSA. The peaks at 1447.94 and 1395.47 cm^-1^ represented the presence of BSA's CH2 bending groups (C-H bending vibration) band. Finally, the peak at 1239.27 cm^-1^ displayed the presence of the amide III bands of BSA (Supplementary Table **S2**).

**Supplementary Table S2.** The shift and/or changes in the wave numbers (cm^-1^) of the characteristic bands of BSA after formulation and preparation of BBR-BSA NPs and BSA NPs

| **Assignation** | **Crystalline BSA**  **(cm^-1^)** | **BBR-BSA NPs**  **(cm^-1^)** | **BSA NPs**  **(cm^-1^)** |
| --- | --- | --- | --- |
| **OH stretching vibration** | 3415.58 | 3381.15 | 3304.05 |
| **Amide A** | 2924.53 | 2926.32 | 2926.09 |
| **Amide B** | 2856.73 | 2860.43 | 2857.37 |
| **Amide I** | 1645.90 | 1653.96 | 1655.34 |
| **Amide II** | 1540.55 | 1538.46 | 1538.62 |
| **CH_2_ bending groups** | (1444.36, 1395.20) | (1448.38, 1391.14) | (1447.94, 1395.47) |
| **Amide III** | 1239.93 | 1236.77 | 1239.27 |

**Supplementary Table S3.** The shift and/or change in the wave numbers (cm^-1^) of the characteristic bands of BBR-chloride after formulation and preparation of BBR-BSA NPs

| **Assignation** | **Standard BBR-chloride**  **(cm^-1^)** | **BBR-BSA NPs**  **(cm^-1^)** |
| --- | --- | --- |
| **OH stretching vibration** | 3407.33 | 3381.15 |
| **Quaternary ammonium (N^+^) group** | 3052.67 | 2926.32 |
| **Saturated C-H stretching** | 2944.96 |  |
| **-OCH_3_ Methoxyl groups** | 2845.13 | 2860.43 |
| **C-N heterocyclic amines** | 1626.49 | 1653.96 |
| **C=N heterocyclic amines** | 1599.69 | 1538.46 |
| **C=C bending vibration** | 1567.57 |  |
| **C=C stretching vibration and**  **furyl groups** | 1505.93 | 1448.38 |
| **-CH2- methylene** | (1479.38-1427.96) |  |
| **-CH3 stretching vibrations** | (1391.74-1362.85) | 1391.14 |
| **C-O-C bonding** | (1333.17-1036.04) | (1236.77-1036.02) |
| **=C-H "OOP" out of plane** | (971.17-730.68) | Encapsulated |
| **Chloride as halide** | (620.60-558.81) | 618.83 |

# Thermal Stability (TGA-DTA/DSC) Analysis:

**a)**

| 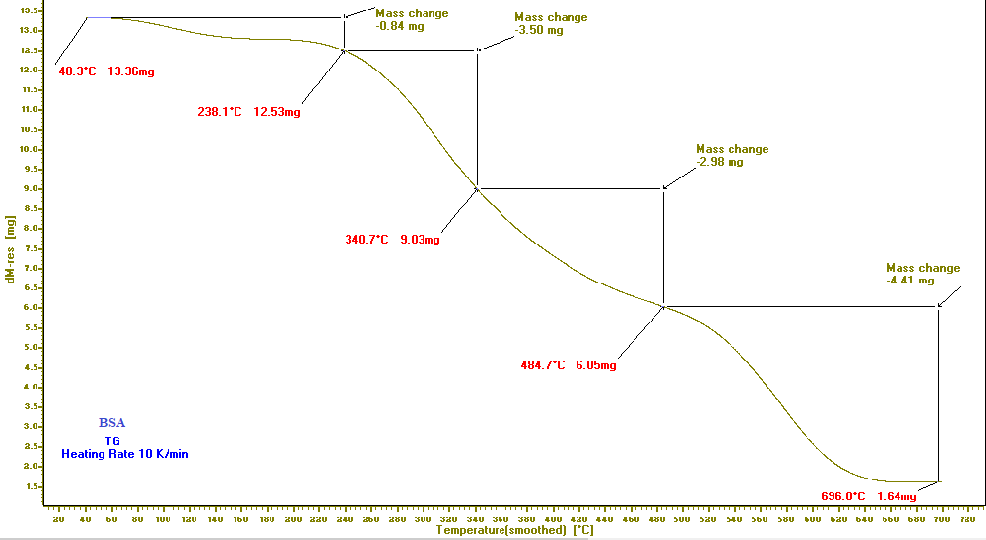  **b)** |
| --- |
| 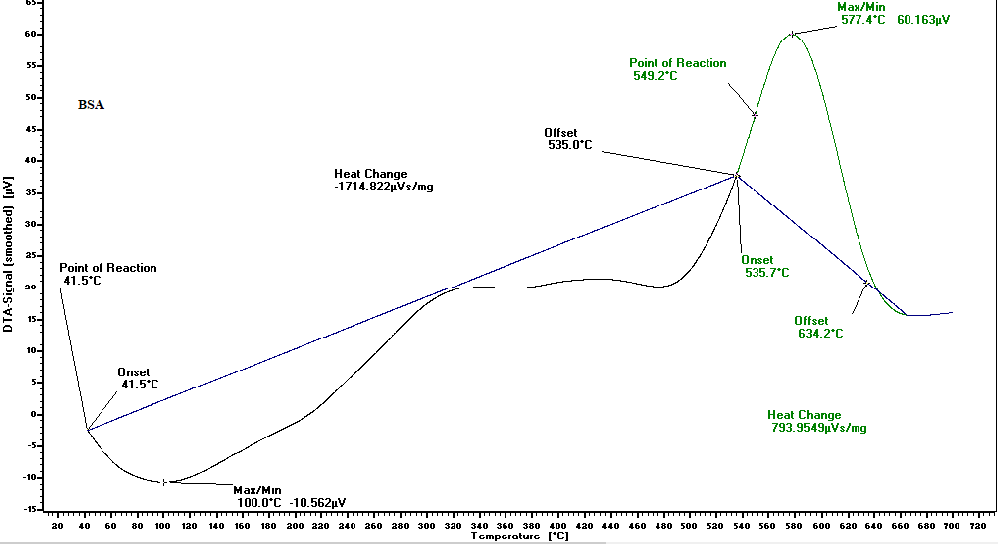  **c)** |
| 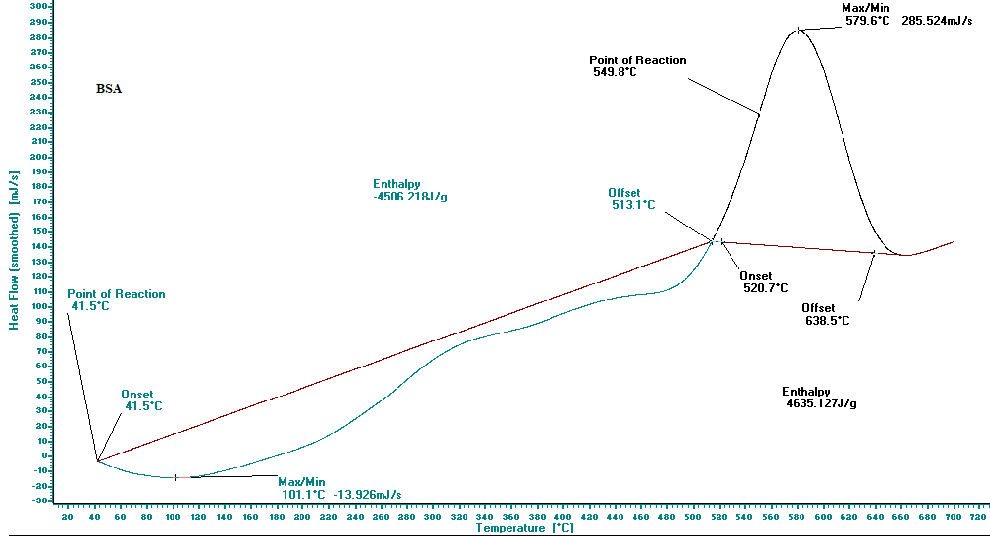 |

Supplementary Figure S1. Thermograms of BSA. TGA (a), DTA (b), and DSC analysis (c).

# Estimation of the free radicals scavenging activities and the total antioxidant capacities of BBR-BSA NPs and BSA NPs

# Nitric Oxide (NO^.^) Scavenging Activity

All diluted compounds, 50 µL at various concentrations, were added to 0.5 mL of 10 mM sodium nitroprusside and 0.45 mL of 0.1 M phosphate-buffered saline (PBS, pH 7.4), which were incubated at 25 ^o^C for 150 min. After incubation, the reaction mixture was mixed with 1 mL of Griess reagent (1% Sulphanilamide, 0.1% naphthylethylenediamine dihydrochloride in 5% H_3_PO_4_) for 30 min at room temperature. A pink-colored chromophore was formed during the diazotization of nitrite ions with sulphanilamide and subsequent coupling with naphthylethylenediamine dihydrochloride. The absorbance of samples was measured at 546 nm against the corresponding sample blank solution (50 µL sample + 0.5 mL sodium nitroprusside + 0.45 mL PBS + 1 mL 5% H_3_PO_4_). Negative control (0.5 mL of 10 mM sodium nitroprusside in PBS and 0.5 mL PBS) was incubated at 25 ^o^C for 150 min. After incubation, the reaction mixture was mixed with 1 mL of Griess reagent for 30 min at room temperature. The absorbance of samples was measured at 546 nm. The blank of the negative control contains 0.5 mL sodium nitroprusside, 0.5 mL PBS and 1 mL 5% H_3_PO_4_.

|  |
| --- |
|  |
|  |

Supplementary Figure S2. NO^.^ radical scavenging activities of (a) standard BBR-chloride (0.01-1.8 mg/mL), BBR-BSA NPs (0.01-1.8 mg BBR/mL) and ascorbic acid (0.01-1.8 mg/mL, reference standard) and (b) BSA (0.5-40 mg BSA/mL) and BSA NPs (0.5-40 mg BSA NPs/mL). (c) Sodium nitrite (NaNO_2_, 5-232 µM) was used as a positive control . Data values are expressed as means ± SD (n = 5).

Supplementary Figure S2 shows that the NO scavenging activity of all the tested compounds increased concentration-dependent. BBR-BSA NPs demonstrated the highest scavenging activity, reaching 68%, followed by ascorbic acid (58%) and BBR (50%), respectively. While BSA and BSA NPs revealed a weak scavenging activity, 21 and 26%, respectively.

# Hydrogen Peroxide (H_2_O_2_) Scavenging Activity

A solution of 300 mM H_2_O_2_ was prepared in 0.1 M phosphate buffer (PB, pH 7.4). An aliquot of different samples with varying concentrations (50 µL), 0.45 mL of 0.1 M PB, and 1.5 mL of 300 mM H_2_O_2_ solution was mixed. The absorbance of the test samples was measured at 230 nm within 10 min against a sample blank solution that contained a reaction mixture without H_2_O_2_ solution (50 µL sample + 1.95 mL 0.1 M PB). The negative control contains 1.5 mL of 300 mM H_2_O_2_ and 0.5 mL of 0.1 M PB against its blank (2 mL of 0.1 M PB).

|  |
| --- |
|  |

Supplementary Figure S3. H_2_O_2_ radical scavenging activities of (a) standard BBR-chloride (0.01-1.8 mg/mL), BBR-BSA NPs (0.01-1.8 mg BBR/mL) and ascorbic acid (0.01-1.8 mg/mL, reference standard) and (b) BSA (0.5-40 mg BSA/mL) and BSA NPs (0.5-40 mg BSA NPs/mL). Data values are expressed as means ± SD (n = 5).

Supplementary Figure S3 demonstrates that the H_2_O_2_ scavenging activity of all the tested compounds was increased in a concentration-dependent manner. BBR-BSA NPs show the highest scavenging activity reaching 95% scavenging, followed by BBR (45%) and ascorbic acid (38%), respectively. While BSA and BSA NPs exhibited a weak H_2_O_2_ scavenging activity, 13 and 23%, respectively.

# Superoxide Anion (O_2_^-^) Scavenging Activity

The O_2_^-^ radicals generated in a negative control tube (PMS/ NADH-NBT system) that include 0.5 mL of 0.3 mM nitroblue tetrazolium (NBT), 0.5 mL of 0.936 mM reduced nicotinamide adenine dinucleotide (NADH), 1.5 mL of 16 mM Tris–HCl buffer (pH 8.0) and 0.2 mL of 0.12 mM phenazine methosulfate (PMS). This mixture was incubated at 25 ^o^C for 5 min, and then the absorbance was measured at 560 nm against a negative control blank (0.5 mL NBT, 0.5 mL NADH, and 1.7 mL Tris-HCl buffer). For test samples, O_2_^-^ radicals generation was inhibited using 0.5 mL NBT, 0.5 mL NADH, 0.1 mL test sample, 1.4 mL Tris buffer and 0.2 mL PMS. The absorbance of the test sample was measured at 560 nm against its sample blank (0.5 mL NBT, 0.5 mL NADH, 0.1 mL test sample, and 1.6 mL Tris-HCl buffer).

|  |
| --- |
|  |

Supplementary Figure S4. O_2_^-^ radical scavenging activities of (a) standard BBR-chloride (0.01-1.8 mg/mL), BBR-BSA NPs (0.01-1.8 mg BBR/mL) and ascorbic acid (0.01-1.8 mg/mL) and (b) BSA (0.5-40 mg BSA/mL) and BSA NPs (0.5-40 mg BSA NPs/mL). Data values are expressed as means ± SD (n = 5).

Supplementary Figure S4 illustrated that the O_2_^-^ scavenging activity of all the tested compounds was increased in a concentration-dependent manner. BBR-BSA NPs demonstrate the highest scavenging activity reaching 64% scavenging, followed by BBR (28%) and ascorbic acid (21%), respectively. While BSA and BSA NPs showed a weak O_2_^-^ scavenging activity, 13 and 21%, respectively.

# Hydroxyl Radical (OH^-^) Scavenging Activity

Ascorbic acid/Fe^3+^/EDTA/H_2_O_2_ was introduced as a Fenton reaction model (hydroxyl radical generation model). The assay was based on the quantification of the 2-deoxy-D-ribose degradation byproducts that introduced a pink chromogen upon heating with thiobarbituric acid (TBA) at low pH. The negative control was included 0.1 mL 2-deoxy-D-ribose (28 mM in 20 mM KH_2_PO_4_-KOH buffer, pH 7.4), 0.5 mL K. phosphate buffer, 0.1 mL of 1.04 mM EDTA, 0.1 mL of 200 µM ferric chloride (FeCl_3_), 0.1 mL of 1.0 mM H_2_O_2_ and 0.1 mL of 1.0 mM ascorbic acid, which incubated at 37 ^o^C for 1 h. One milliliter of 1% TBA in 0.25 N HCl and 1.0 mL of 2.8% trichloroacetic acid (TCA) were added to the mixture, which was incubated in a boiling water bath for 20 min and centrifuged at 3000 rpm for 20 min. After cooling, the absorbance of the supernatant was measured at 532 nm against a negative control blank (as in previous steps except for the first step that added buffer instead of 2-deoxy-D-ribose). For all test samples, as in previous steps, except at the beginning was added 0.1 mL 2-deoxy-D-ribose, 0.1 mL sample, and 0.4 mL buffer were against the sample blank that was added at the beginning, 0.1 mL sample and 0.5 mL buffer.

|  |
| --- |
|  |

Supplementary Figure S5. OH^-^ radical scavenging activities of (a) standard BBR-chloride (0.01-1.8 mg/mL), BBR-BSA NPs (0.01-1.8 mg BBR/mL) and ascorbic acid (0.01-1.8 mg/mL) and (b) BSA (0.5-40 mg BSA/mL) and BSA NPs (0.5-40 mg BSA NPs/mL). Data values are expressed as means ± SD (n = 5).

Supplementary Figure S5 illustrated that the OH^-^ scavenging activity of all the tested compounds was increased in a concentration-dependent manner. BBR-BSA NPs demonstrated the highest scavenging activity reaching 99% scavenging, followed by BBR (94%) and ascorbic acid (67%), respectively. While BSA and BSA NPs showed a weak O_2_^-^ scavenging activity, 28 and 41%, respectively.

# 1, 1-diphenyl-2-picrylhydrazyl (DPPH) Scavenging Activity

Briefly, 0.3 mM DPPH methanolic solution was prepared. Furthermore, 0.8 mL of this solution was added to 1.2 mL of 0.1 M Tris-HCl (pH 7.4) to give a negative control. Moreover, 0.8 mL of methanol and 1.2 mL of Tris-HCl were considered as a blank of the negative control. For different samples, 0.8 mL of 0.3 mM DPPH solution, 1150 µL of Tris-HCl, and 50 µL of the test sample were added. The sample blank included 0.8 mL of methanol, 1150 µL of Tris-HCl, and 50 µL of the test sample. The reaction mixture was mixed for 10 s and left to stand at room temperature in a dark place for 2 min. The absorbance was measured at 517 nm using a UV scanning spectrophotometer against the corresponding blank.

|  |
| --- |
|  |

Supplementary Figure S6. DPPH radical scavenging activities of (a) standard BBR-chloride (0.01-1.8 mg/mL), BBR-BSA NPs (0.01-1.8 mg BBR/mL) and ascorbic acid (0.01-1.8 mg/mL, reference standard) and (b) BSA (0.5-40 mg BSA/mL) and BSA NPs (0.5-40 mg BSA NPs/mL). Data values are expressed as means ± SD (n = 5).

Supplementary Figure S6 illustrated that the DPPH scavenging activity of all the tested compounds increased concentration-dependent. BBR-BSA NPs demonstrated the highest scavenging activity reaching 99% scavenging, followed by BBR (95%) and ascorbic acid (78%), respectively. While BSA and BSA NPs showed a weak O_2_^-^ scavenging activity, 31 and 40%, respectively.

# Thiobarbituric Acid Reactive Substances (TBARS) Scavenging Activity

First, 250 µL of tissue homogenate supernatant (lipid source) and 100 µL of different diluted samples, reference standard or PB (negative control) were added to a dry test tube that made up to 500 µL with a PB. This mixture was incubated at 37 ^o^C for 1 h. Then, 100 µL of 0.075 M ferrous sulphate (FeSO_4_) and 20 µL of 0.1 M ascorbic acid were added, mixed, and incubated for 1 h at 37 ^o^C to induce the generation of the lipid peroxides in the liver and brain tissue homogenates. Furthermore, 200 µL of 0.1 M EDTA was also added to terminate this process as a preventive antioxidant agent (transient metal chelator). Moreover, 1mL of 15% TCA was added, mixed, and centrifuged into the tubes at 4000 rpm for 20 min. The supernatant was taken and mixed with 1 mL of 1% TBA in 0.25 N HCl to heat for 20 min at 100 ^o^C. After cooling, the absorbances of the different tubes were measured at 532 nm against their blank tubes (all previous steps except the first step, which added PB instead of tissue homogenate extract).

|  |
| --- |
|  |
|  |
|  |

Supplementary Figure S7. TBARS radical scavenging activities of standard BBR-chloride (0.01-1.8 mg/mL), BBR-BSA NPs (0.01-1.8 mg BBR/mL) and ascorbic acid (0.01-1.8 mg/mL, reference standard) in liver (a) and brain tissue homogenates (c). Effects of BSA (0.5-40 mg BSA/mL) and BSA NPs (0.5-40 mg BSA NPs/mL) in liver (b) and brain tissue homogenate (d) were represented. Data values are expressed as means ± SD (n = 5).

Supplementary Figure S7 illustrated that the liver and brain TBARS scavenging activity of all the tested compounds increased concentration-dependent. BBR-BSA NPs demonstrated the highest scavenging activity, followed by BBR and ascorbic acid. While BSA and BSA NPs showed a weak O_2_^-^ scavenging activity. Moreover, the NPs formulations of both BBR and BSA were found to increase the radical scavenging activity more than the free compound.

# Total antioxidant capacities (TAC)/ Ferric Reducing Power (FRP)

For all diluted samples, 0.1 mL of sample was added to 0.5 mL of 0.2 M PB (pH 6.6) and 0.5 mL of 1% potassium ferricyanide [K_3_Fe (CN)_6_], which were incubated at 50 °C for 20 min in a hot water bath. Then, 0.5 mL of 10% TCA was added to the reaction mixture and centrifuged at 3000 rpm for 10 min. Finally, 1 mL of the upper layer of the solution was mixed with 1 mL of distilled water and 0.5 mL of 0.1% FeCl_3_. The absorbance of the resulted sample solution (Perl's Prussian blue color) was measured at 700 nm after 5 min at room temperature against a sample blank (all previous reaction steps except the final step, which added 0.5 mL of PB instead of FeCl_3_). The negative control was prepared through all previous steps except the first step, which added 0.1 mL of PB instead of the sample against its blank without the FeCl_3_ step. A higher absorbance indicated a greater reducing capacity.

|  |
| --- |
|  |

Supplementary Figure S8. Total antioxidant capacities (TAC). Ferric reducing powerful (FRP) of (a) standard BBR-chloride (0.01-1.8 mg/mL), BBR-BSA NPs (0.01-1.8 mg BBR/mL) and ascorbic acid (0.01-1.8 mg/mL, reference standard) and (b) BSA (0.5-40 mg BSA/mL) and BSA NPs (0.5-40 mg BSA NPs/mL). Data values are expressed as means ± SD (n = 5).

Supplementary Figure S7 demonstrates that the ferric reducing power of all tested compounds increased proportionally to their concentration. BBR-BSA NPs exhibited more significant reducing activity than BBR. While BSA and BSA NPs exhibited a weak ferric reducing power, BSA and BSA NPs did not. In addition, it was discovered that the NPs formulations of both BBR and BSA increased the ferric reducing power compared to the free compounds.

**Total antioxidant capacities (TAC)**/ green phosphomolybdenum method

The target sample (0.1 mL) was mixed with the phosphomolybdenum complex reagent (1 mL, 28 mM sodium phosphate and 4 mM ammonium molybdate in 0.6 M sulfuric acid solution). The tube was capped and incubated in a boiling water bath at 95 ^o^C for 90 min. After cooling, the absorbance of the aqueous solution was measured at 695 nm against its blank. The sample blank included 1 mL of the reagent and an appropriate volume of the sample solvent, which was incubated under the same conditions. For negative control, 0.1 mL of PB was included instead of the test sample.

|  |
| --- |
|  |

Supplementary Figure S9. Total antioxidant capacities (TAC). Green phosphomolybdenum complex of (a) standard BBR-chloride (0.01-1.8 mg/mL), BBR-BSA NPs (0.01-1.8 mg BBR/mL) and ascorbic acid (0.01-1.8 mg/mL, reference standard) and (b) BSA (0.5-40 mg BSA/mL) and BSA NPs (0.5-40 mg BSA NPs/mL). Data values are expressed as means ± SD (n = 5).

Green phosphomolybdenum complex was formed due to the tested compounds' total antioxidant potentials. Supplementary Figure S9 illustrated that the density of the green phosphomolybdenum complex was increased by increasing the concentrations of the tested compounds. BBR-BSA NPs showed higher antioxidant capacity than BBR. While BSA and BSA NPs showed a weak antioxidant capacity. Furthermore, the NPs formulations of both BBR and BSA were found to increase the total antioxidant capacity than the free compound.

# Red Blood Cells Lysis Rate

Ten milliliters of human blood were placed in trisodium citrate or K_2_-EDTA-coated vacutainer tubes to prevent coagulation. The collected human blood sample was centrifuged at 4000 rpm for 5 min at 4 ^o^C. The levels of the hematocrit (red, lower layer) and plasma (yellowish, upper layer) were marked on the tubes (37% hematocrit). The plasma and the buffy coat were gently aspirated via a micropipettor. Then, the hematocrit tubes were filled to the original plasma levels with sterilized Mcllvaine's buffer. The latter hematocrit tubes were covered and inverted a few times to mix and centrifuge at 4000 rpm for 5 min at 4 ⁰C. The red blood cell washing step was repeated 3 to 5 times. Furthermore, the supernatant was aspirated, and the red blood cells were diluted with the sterilized Mcllvaine's buffer, which became 4% hematocrit. For the positive control sample, 10 μL of 40% Triton X-100 (100% hemolysis) was used. For the negative control sample, 200 μL of Mcllvaine's buffer was added to 1 mL of the diluted erythrocytes (4% hematocrit), which were incubated for one hour at 37 °C in a shaker. One milliliter of the diluted erythrocytes was incubated for one hour at 37 °C in a shaker with 200 µL of the different samples, reference standard, H_2_O, or 300 mM H_2_O_2_. The previous tubes were centrifuged for 10 min at 7000 rpm to obtain the supernatants. The amount of the liberated hemoglobin in the supernatant was measured at 540 nm against air.

|  |
| --- |
|  |

Supplementary Figure S10. Red blood cells (RBCs) lysis rates (%). Anti-hemolytic powerful of (a) standard BBR-chloride (0.01-1.8 mg/mL), BBR-BSA NPs (0.01-1.8 mg BBR/mL) and ascorbic acid (0.01-1.8 mg/mL, reference standard) and (b) BSA (0.5-40 mg BSA/mL) and BSA NPs (0.5-40 mg BSA NPs/mL). Data values are expressed as means ± SD (n = 5).

Supplementary Figure S10 illustrated that the tested compounds had potent anti-hemolytic activities within less than 5% hemolysis. BBR-BSA NPs demonstrated the most significant anti-hemolytic activities, followed by BSA NPs, ascorbic acid, BBR and BSA, respectively

# Activated Partial Thromboplastin Time (APPT)

Citrated human plasma was prepared by mixing the normal human blood with 3.8% trisodium citrated (9:1, v/v) tubes, then centrifuged at 3000 rpm for 10 min to obtain the platelet-poor plasma (PPP). Furthermore, 50 µL of the PPP and 10 µL of the different diluted tested samples [BBR, BBR-BSA NPs, and ascorbic acid (0.01-1.8 mg/mL); BSA and BSA NPs (0.5-40 mg/mL)] were added and incubated for 20 min at the room temperature. Moreover, 50 µL of the APTT reagent was added and was incubated at 37 ^o^C for an additional 5 min. Afterward, 50 µL of 0.02 M pre-incubated calcium chloride (CaCl2) at 37 oC was delivered to initiate the clotting reaction. In addition, 10 µL of milli-Q H_2_O or Mcllvaine's buffer was used instead of the tested samples to evaluate the coagulation time (sec) as a negative control.

|  |
| --- |
|  |

Supplementary Figure S11. Activated partial thromboplastin time (APTT). Anti-clotting properties of (a) standard BBR-chloride (0.01-1.8 mg/mL), BBR-BSA NPs (0.01-1.8 mg BBR/mL) and ascorbic acid (0.01-1.8 mg/mL, reference standard) and (b) BSA (0.5-40 mg BSA/mL) and BSA NPs (0.5-40 mg BSA NPs/mL). Data values are expressed as means ± SD (n = 5).

Supplementary Figure S11 revealed that the anti-clotting potentials of all the tested compounds were increased in a concentration-dependent manner. BBR-BSA NPs demonstrated the highest anti-clotting potential reaching 93 sec, followed by ascorbic acid (83 sec) and BBR (79 sec), respectively. While BSA and BSA NPs showed a weak anti-clotting potential, 59 and 68 sec, respectively.

Supplementary Table S4. The free radicals scavenging activities, total antioxidant capacities, anti-hemolytic capacity, and anti-clotting properties of the tested compounds.

| **Formulations**  **Antioxidant potentials** | **BBR**  **(1.8 mg/mL)** | **BBR-BSA NPs**  **(1.8 mg/mL)** | **Ascorbic acid**  **(1.8 mg/mL)** | **BSA**  **(40 mg/mL)** | **BSA NPs**  **(40 mg/mL)** |
| --- | --- | --- | --- | --- | --- |
| **NO^.^ radical scavenging (%)^1^** | 50.22 ± 2.66^a^ | **68.77 ± 2.66^b^** | 58.22 ± 1.81^c^ | 21.33 ± 1.07^d^ | 26.33 ± 0.81^e^ |
| **H_2_O_2_ radical scavenging (%)** | 45.35 ± 1.33^a^ | **94.71 ± 2.61^b^** | 37.71 ± 1.52^c^ | 12.88 ± 0.68^d^ | 22.95 ± 1.17^e^ |
| **O_2_^-^ radical scavenging (%)** | 27.82 ± 0.58^a^ | **64.22 ± 2.44^b^** | 20.86 ± 0.66^c^ | 13.11 ± 0.57^d^ | 20.63 ± 0.52^e^ |
| **OH- radical scavenging (%)** | 93.66 ± 2.62^a^ | **98.81 ± 1.19^b^** | 67.33 ± 1.42^c^ | 28.11 ± 1.17^d^ | 41.22 ± 2.11^e^ |
| **DPPH radical scavenging (%)** | 95.19 ± 2.43^a^ | **98.91 ± 1.58^a^** | 77.67 ± 2.16^b^ | 31.11 ± 1.77^c^ | 39.65 ± 1.62^d^ |
| **Liver TBARS radical scavenging (%)** | 77.72 ± 1.82^a^ | **97.44 ± 2.48^b^** | 81.94 ± 2.46^c^ | 28.11 ± 1.12^d^ | 39.71 ± 1.48^e^ |
| **Brain TBARS radical scavenging (%)** | 77.49 ± 1.69^a^ | **90.11 ± 2.22^b^** | 66.69 ± 2.21^c^ | 25.11 ± 1.35^d^ | 34.77 ± 1.35^e^ |
| **TAC/ FRP^2^** | 0.483 ± 0.032^a^ | **0.711 ± 0.041^b^** | 1.147 ± 0.072^c^ | 0.133 ± 0.011^d^ | 0.268 ± 0.012^e^ |
| **TAC/ Green Phosphomolybdenum^2^** | 0.584 ± 0.023^a^ | **0.695 ± 0.044^b^** | 1.123 ± 0.061^c^ | 0.181 ± 0.013^d^ | 0.256 ± 0.013^e^ |
| **RBCs lysis rates (%)^3^** | 3.11 ± 0.144^a^ | **0.47 ± 0.011^b^** | 2.79 ± 0.163^c^ | 4.37 ± 0.211^d^ | 1.29 ± 0.088^e^ |
| **APPT (sec)** | 79.4 ± 0.9^a^ | **92.7 ± 0.8^b^** | 82.9 ± 1.1^c^ | 58.8 ± 0.6^d^ | 67.7 ± 1.2^e^ |

NO^.^, nitric oxide; H_2_O_2_, hydrogen peroxide; O_2_^-^, superoxide anion; OH-, hydroxyl radical; DPPH, 1, 1-diphenyl-2-picrylhydrazyl; TBARS, thiobarbituric acid reactive substances; TAC, total antioxidant capacities; FRP, ferric reducing power; RBCs, red blood cells; APPT, activated partial thromboplastin time.

**^1^** Sodium nitrite (NaNO_2_) as a positive control inhibited 45.81 ± 2.19% NO^.^ at 232 μM with IC_50_ or EC_50_ value of 256.34 ± 3.11 μM.

**^2^** Negative control absorbance was 0.054 and 0.096 for FRP and green Phosphomolybdenum complex, respectively.

**^3^** 10 μL of 40% Triton X-100 caused 100% hemolysis, while 200 µL of H_2_O or 300 mM H_2_O_2_ as a standard radical's source introduced 1.4 or 1.1% hemolysis, respectively.

Data values are expressed as means ± SD (n = 5). Different letters in the same row indicated statistically signiﬁcant differences (*p* < 0.05) that were evaluated using the analysis of one-way ANOVA with *a post hoc* LSD test. The same letters in the same row indicated non-signiﬁcant differences (*p* ˃ 0.05).

# Antimicrobial Activity

Supplementary Table S5. The antimicrobial activities of BSA and BSA NPs (0.5-40 mg/mL)

| **Concs (mg/mL)** | **Zones of inhibition (mm)** | | | | | | | |
| --- | --- | --- | --- | --- | --- | --- | --- | --- |
|  | **BSA** | | | | **BSA NPs** | | | |
|  | ***E. coli*** | ***C. albicans*** | ***B. subtilis*** | ***S. aureus*** | ***E. coli*** | ***C. albicans*** | ***B. subtilis*** | ***S. aureus*** |
| **0.5** | NE | NE | NE | NE | NE | NE | NE | NE |
| **1** | NE | NE | NE | NE | NE | NE | NE | NE |
| **5** | NE | NE | NE | NE | NE | NE | NE | NE |
| **10** | NE | NE | NE | NE | NE | NE | NE | NE |
| **15** | NE | NE | NE | NE | NE | NE | NE | NE |
| **20** | NE | NE | NE | NE | NE | NE | NE | NE |
| **25** | NE | NE | NE | NE | NE | NE | NE | NE |
| **30** | NE | NE | NE | NE | NE | NE | NE | NE |
| **40** | NE | NE | NE | NE | NE | NE | NE | NE |

NE, non-effected

Supplementary Table S6. The antimicrobial activities of BBR and BBR-BSA NPs (0.01-1.8 mg/mL)

| **Concs (mg/mL)** | **Zones of inhibition (mm)** | | | | | | | |
| --- | --- | --- | --- | --- | --- | --- | --- | --- |
|  | **BBR** | | | | **BBR-BSA NPs** | | | |
|  | ***E. coli*** | ***C. albicans*** | ***B. subtilis*** | ***S. aureus*** | ***E. coli*** | ***C. albicans*** | ***B. subtilis*** | ***S. aureus*** |
| **0.01** | ND | 2.3 ± 0.2 | ND | ND | 2.3 ± 0.6 | 2.5 ± 0.4 | 2.9 ± 0.7 | 2.4 ± 0.3 |
| **0.05** | ND | 7.6 ± 0.9 | ND | ND | 6.6 ± 0.8 | 9.3 ± 1.1 | 9.7 ± 0.9 | 9.4 ± 1.1 |
| **0.1** | 7.5 ± 1.2 | 8.8 ± 1.1 | 5.8 ± 0.6 | 9.5 ± 1.2 | 10.5 ± 1.2 | 9.8 ± 1.7 | 11.5 ± 1.5 | 12.3 ± 1.6 |
| **0.25** | 12.3 ± 2.3 | 13.3 ± 2.1 | 14.3 ± 1.3 | 10.3 ± 1.6 | 15.5 ± 0.9 | 15.7 ± 0.9 | 15.5 ± 1.4 | 16.7 ± 2.1 |
| **0.5** | 16.6 ± 1.4 | 18.6 ± 1.2 | 18.7 ± 1.1 | 15.9 ± 1.7 | 19.8 ± 2.3 | 19.9 ± 1.4 | 19.7 ± 2.7 | 20.7 ± 2.4 |
| **1** | 21.8 ± 1.8 | 21.8 ± 2.2 | 22.3 ± 1.6 | 22.8 ± 2.3 | 26.4 ± 1.9 | 23.3 ± 2.5 | 23.4 ± 1.2 | 25.4 ± 1.6 |
| **1.5** | 25.3 ± 1.4 | 25.3 ± 1.8 | 24.8 ± 2.6 | 27.8 ± 2.7 | 29.7 ± 1.9 | 29.4 ± 1.3 | 26.4 ± 1.2 | 30.4 ± 1.6 |
| **1.8** | 29.3 ± 1.5 | 27.8 ± 1.4 | 26.6 ± 1.2 | 30.8 ± 1.7 | 32.7 ± 1.3 | 31.3 ± 1.7 | 30.8 ± 2.2 | 32.7 ± 1.3 |

ND, non-detected

| 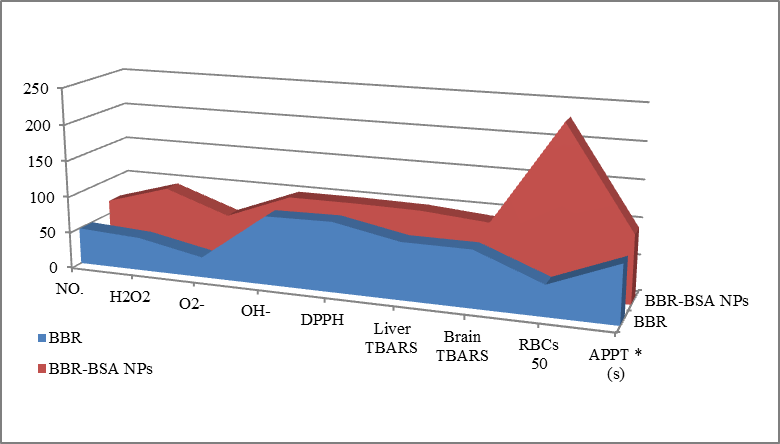  **a)** | 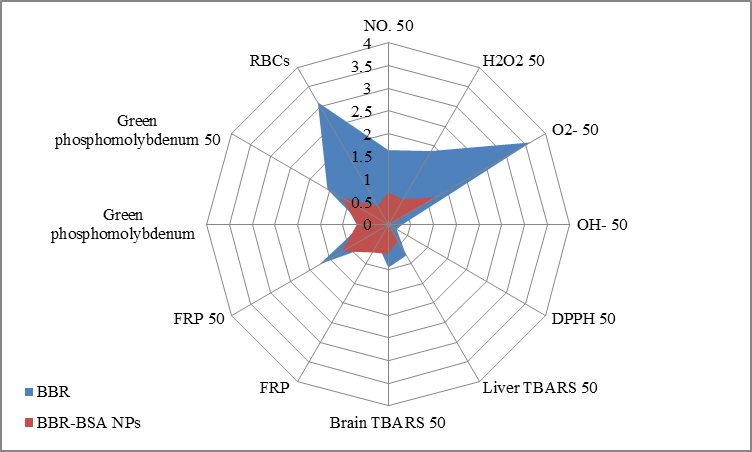  **b)** |
| --- | --- |
| 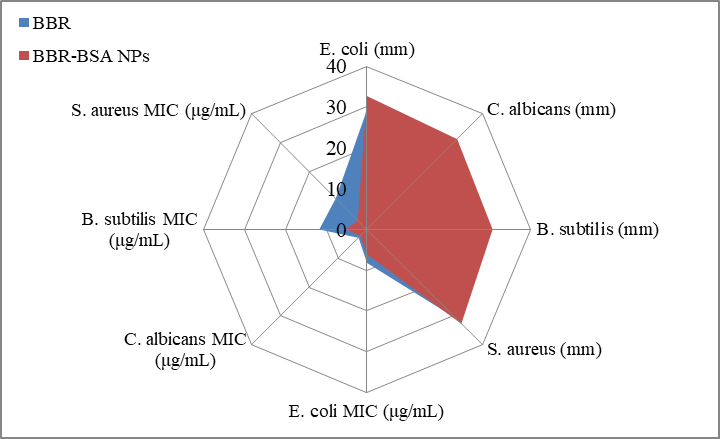  **c)** | 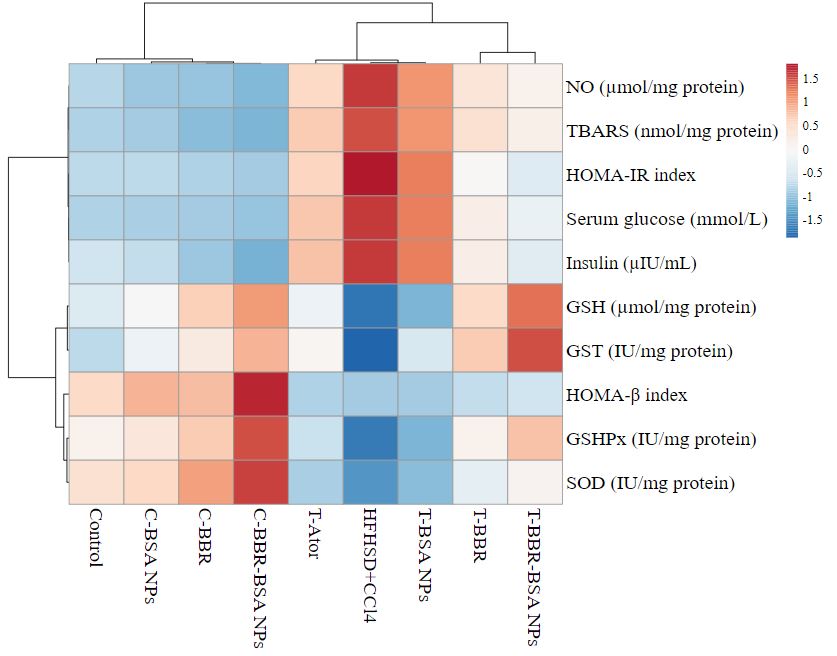  **d)** |

Supplementary Figure S12. The in vitro anti-radical and antioxidant potentials (a, b) and antimicrobial activities (c) of BBR and BBR-BSA NPs. ClustVis heatmap distribution analysis of the *in vivo* biochemical, oxidative stress, and antioxidant parameters according to the variations within the different experimental groups (d) (<http://biit.cs.ut.ee/clustvis/>). The red color indicated an increase in the values of the target parameters, while the blue color demonstrated a decrease in the values of these parameters.
